# Predictors of post-retirement mortality in athletes required to maintain very high body mass: a retrospective cohort study using AI-driven three-dimensional body reconstruction, 1865–1990

**DOI:** 10.7717/peerj.21924

**Published:** 2026-11-10

**Authors:** Akira Kimura

**Affiliations:** Department of Physical Therapy, Graduate School of Health Sciences, Gunma Paz University, Takasaki, Gunma, Japan

**Keywords:** Sumo wrestlers, Super-obesity, Post-retirement mortality, Historical cohort study, Immortal time bias, Standardised mortality ratio, AI-based body composition, Central obesity, Occupational mortality

## Abstract

**Background:**

Elite athletes in weight-uncapped disciplines sustain very high body mass throughout their careers (body mass index (BMI) 35–50 kg/m^2^) and show a two- to three-fold mortality excess, yet no post-retirement care framework exists. Prior sumo mortality analyses used career-entry as follow-up origin, introducing immortal time bias. We generated virtual three-dimensional body reconstructions from historical photographs using artificial intelligence (AI)-driven landmark extraction to examine whether a reconstructed waist circumference adds prognostic information on post-retirement mortality beyond BMI.

**Methods:**

All wrestlers reaching the makuuchi division (1865–1990 birth cohort; source *N* = 978) were assembled from published compendia. Transverse abdominal diameter was measured on career-peak photographs calibrated against recorded height; sagittal diameter was recovered by a volume-conserving solver; virtual waist circumference (C3) was the perimeter of the resulting ellipse. Retirement-anchored Cox models adjusted for birth year, retirement age and rank entered one body-size measure at a time; cause-specific models used follow-up from 1990 with multiple imputation of undocumented causes. Standardised mortality ratios (SMRs) used age × calendar-year-specific Japanese male rates. Primary cohort: birth < 1970 (*n* = 604; 587 with a calibrated photograph).

**Results:**

The primary cohort had 467 deaths over median 31.1 years follow-up; median BMI 36.2 kg/m^2^, median C3 136 cm. C3 rose with rank and correlated with BMI (*r* = 0.95). C3 per standard deviation (SD) (hazard ratio (HR) 1.47, 95% confidence interval (CI) [1.29–1.69]; Harrell’s C 0.675) and BMI per SD (HR 1.38, 1.22–1.55; C 0.672) showed similar prognostic performance and could not be separated. The photographic diameter alone did not predict mortality (HR 1.08, 0.95–1.22) and added nothing to BMI (HR 1.03). Elite rank (HR 1.08) and career length (HR 0.98) showed no association. From 1990, with imputation, BMI predicted cardiometabolic death (HR 1.42, 1.08–1.87) but not neoplastic death (HR 1.07, 0.73–1.56), and C3 showed the same pattern (cardiometabolic HR 1.50, 1.08–2.07; neoplastic HR 1.20, 0.77–1.86); elite rank predicted neither. SMRs were 1.89 (1.72–2.07) overall, 1.84 for pre-1950 births and 2.57 for post-1950 births.

**Discussion:**

Sumo wrestlers experienced about twofold all-cause mortality versus matched Japanese males across all historical strata. A waist circumference reconstructed from photographs, height and weight predicted mortality—specifically cardiometabolic mortality—as well as BMI, but its prognostic information derived from recorded body mass: the photographic component added none. Because obesity onset was unrecorded, the reconstruction is unvalidated, and no post-retirement data exist, the null findings for career length and photographic shape are not evidence that obesity duration or abdominal fat distribution is unrelated to mortality.

## Introduction

Adult obesity affects more than one billion people worldwide, with a small but growing subset reaching class III obesity (BMI ≥ 40 kg/m^2^) and increasingly drawn into elite professional strength and grappling sports that systematically reward extreme body size (GBD 2021 Adult BMI Collaborators, 2025; NCD Risk Factor Collaboration, 2024; Rubino et al., 2025). For these athletes—including American football linemen, heavyweight powerlifters and weightlifters, and Japanese professional sumo wrestlers—very high body mass is not an incidental comorbidity but an occupational requirement of the sport itself, sustained throughout the competitive career and frequently into retirement (Nishizawa et al., 1976; Kanehisa et al., 1998; Heshmati, 2025). Despite this, we identified no standardised post-retirement care framework specifically designed for the de-escalation of body mass, cardiometabolic surveillance, or psychological transition of such athletes after career end, leaving them dependent on individual judgement in the face of substantial accumulated risk (Runacres, Mackintosh & McNarry, 2021; Lemez & Baker, 2015).

Mortality studies of retired weight-uncapped athletes have demonstrated two- to three-fold excess cardiovascular and neurodegenerative mortality across multiple disciplines and time periods, supporting biological plausibility of a generalised long-term hazard (Baron et al., 2012; Lehman et al., 2012; Nguyen et al., 2019; Venkataramani, Gandhavadi & Jena, 2018; Sarna et al., 1993; Kujala et al., 1996; Pärssinen et al., 2000; Herman et al., 2014). Before these contemporary studies, smaller obituary-based and cross-sectional analyses of Japanese sumo wrestlers had likewise reported elevated cardiometabolic morbidity and reduced life expectancy, but the inability to extract granular anthropometric data from historical photographic archives—together with the use of career-entry as the follow-up origin, which introduces immortal time bias because reaching the elite division requires several years of survival in pre-elite competition—has hampered separation of elite-rank, era, and anthropometric associations with risk in cohorts long enough to evaluate secular change (Hoshi & Inaba, 1995; Kanda et al., 2009; Nishizawa et al., 1976). These associations are biologically plausible: career-induced obesity entails chronic positive energy balance, visceral adiposity, insulin resistance, hypertension, and accelerated cardiovascular ageing, compounded by repeated musculoskeletal injury and post-competition lifestyle dysregulation that together impose cumulative metabolic and somatic burden (Pi-Sunyer, 2009; Lavie, Milani & Ventura, 2009; Kivimäki et al., 2022; Nyberg et al., 2026).

Existing mortality evidence for weight-uncapped elite athletes is restricted to comparatively short historical spans: the NIOSH American football cohort covers approximately 60 years (Baron et al., 2012; Lehman et al., 2012), the Finnish heavyweight weightlifter cohort 45 years (Sarna et al., 1993; Kujala et al., 1996; Pärssinen et al., 2000), and the contemporary professional wrestling cohort 26 years (Herman et al., 2014). For Japanese professional sumo, the largest prior published study covered 430 wrestlers entering the top division between 1926 and 1989 and used a career-entry follow-up origin, with no prior cohort applying artificial intelligence to historical photographic archives to recover three-dimensional body-shape information at population scale, and no prior analysis of a heavyweight athletic population using age- and calendar-year-stratified reference mortality for SMR estimation. To address these evidence gaps, we developed a hybrid AI–physics pipeline that combines height-calibrated measurement of career-peak photographs with a volume-conserving reconstruction of a virtual three-dimensional waist circumference, and applied it to the full population of 978 Japanese professional sumo wrestlers reaching the top division from the 1865–1990 birth cohort with retirement-anchored follow-up to 2025. We further combined these estimates with age- and calendar-year-stratified Japanese male reference mortality derived from the IPSS Japan Mortality Database to test whether the historically reported mortality excess in this athletic population has changed in line with the secular decline in general-population mortality across the documented period. The analysis is prognostic: it identifies career-period characteristics associated with post-retirement mortality and examines whether the reconstructed waist circumference, and the photographic measurement on which it rests, add prognostic information beyond BMI; it does not estimate causal effects.

## Materials & Methods

### Study design and participants

In this retrospective cohort study, we assembled all wrestlers reaching the makuuchi division of the Japan Sumo Association—the highest competitive division of professional sumo, in which divisions are determined solely by competitive record and there are no weight classes—during the Meiji (1868–1912), Taishō (1912–1926), Shōwa (1926–1989), and early Heisei (1989–2019) eras from published Japanese-language biographical compendia (source cohort *N* = 978). Inclusion required verified career-peak height and weight, a career-peak portrait photograph suitable for landmark extraction, a verified birth year, a verified retirement year, and ascertainable life status. After exclusion of records flagged outside the main analytical set (*n* = 124) and records with missing values on any of the required variables (*n* = 87), the analytical cohort comprised 767 wrestlers; the primary cohort (birth year <1970; *n* = 604) served as the primary analysis to avoid confounding by late-twentieth-century photographic-style change, and wrestlers born 1970 or later (*n* = 163) were analysed as a sensitivity cohort. Analyses involving C3 used the wrestlers for whom a height-calibrated photographic measurement was available (C3 analysis set: 587 of the 604 primary-cohort wrestlers, 450 deaths; 737 of the 767 overall). The full participant flow is shown in Fig. S1.

### Ethics statement

This study did not require ethical approval because all data were drawn from published biographical sources documenting public figures (professional sumo wrestlers in the makuuchi division), and individual-level information is reported only in aggregate at the cohort and subgroup level. Per the Gunma Paz University Research Ethics Committee guidelines, studies based solely on publicly available historical biographical data on public figures are exempt from review.

### Procedures

Career-peak portrait photographs were digitised at 300 dpi from biographical compendia and processed through a hybrid AI–physics pipeline (Fig. 1). Portraits were located and cropped automatically, and the wrestler’s outline and skeletal landmarks were extracted using MediaPipe Pose v0.10 (Google) and YOLOv8 (Ultralytics). The photographic scale (cm per pixel) was obtained for each portrait by dividing the wrestler’s recorded standing height by his height in the photograph in pixels, which for these standing full-length portraits was taken as the height of the portrait frame. The transverse abdominal diameter (TAD) was the maximum horizontal width of the abdominal outline at the umbilicus level in pixels multiplied by this scale. Because the sagittal abdominal diameter (SAD) cannot be observed from a frontal photograph, it was recovered by a volume-conserving solver. The trunk was modelled as a barrel-shaped stack of elliptical cross-sections of length 0.288  × standing height whose volume equals trunk mass divided by trunk density (1.03 g/cm^3^) (Dempster, 1955; Drillis & Contini, 1966; Winter, 2009). For the general adult population the trunk accounts for 0.497 of body mass (Dempster, 1955; Winter, 2009); because the mass of sumo wrestlers is concentrated in a protuberant abdomen, the primary analysis used a trunk mass fraction of 0.55 and took the cross-sectional area at maximal abdominal girth to be 1.20 times the mean cross-sectional area of the trunk. SAD then follows in closed form from the area at maximal girth, (*π*/4) × TAD × SAD. The photograph thus fixes the transverse width, and recorded mass and height fix the depth; the general-population values and intermediate assumptions were examined in sensitivity analyses.

**Figure 1:**
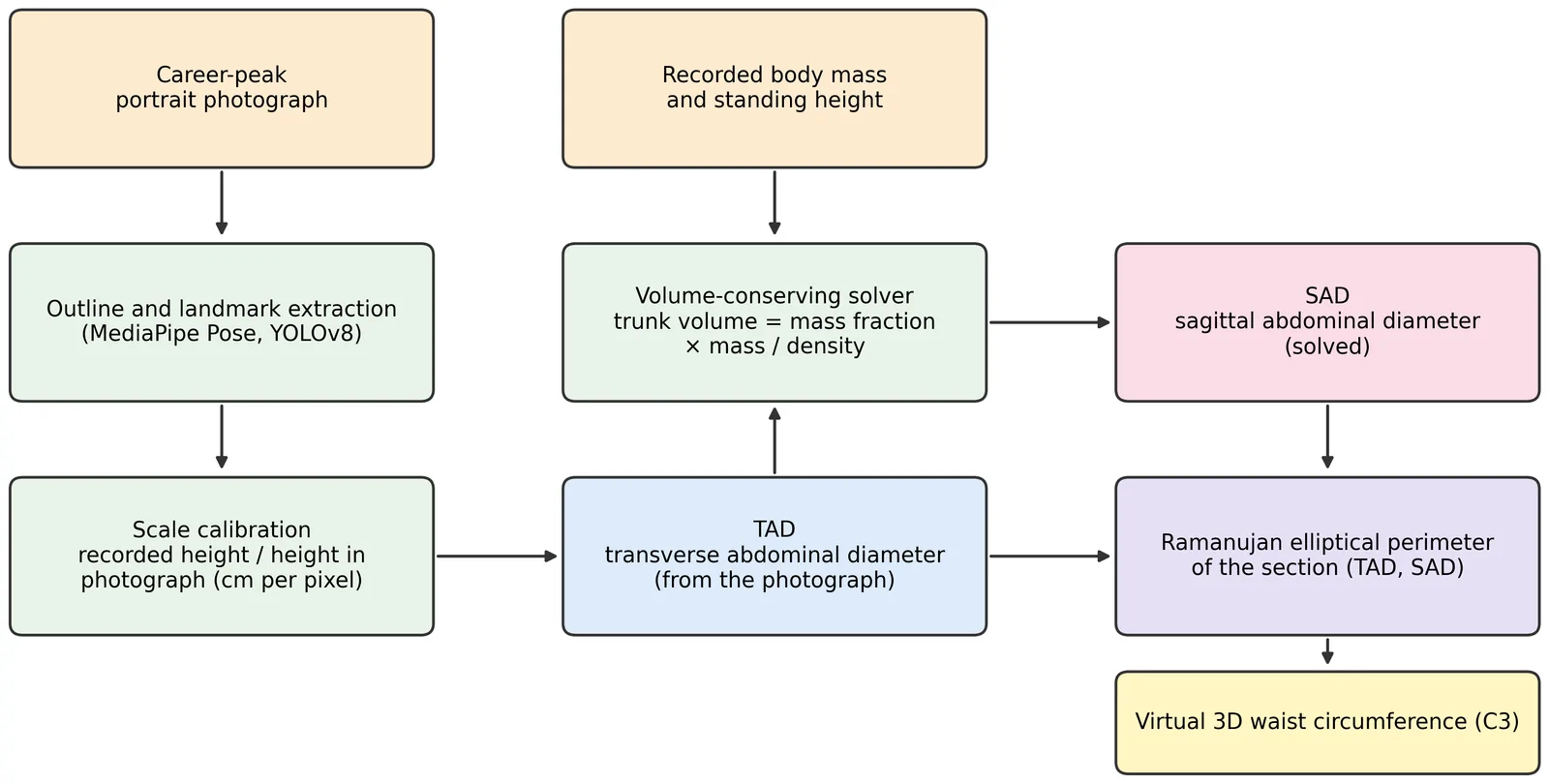
Hybrid AI–physics pipeline for virtual three-dimensional waist circumference reconstruction from career-peak photographs. Workflow overview: career-peak portrait photograph → AI-derived outline and skeletal landmark extraction (MediaPipe Pose, YOLOv8) → photographic-scale calibration against recorded standing height → transverse abdominal diameter (TAD, measured on the calibrated photograph); recorded body mass and standing height → volume-conserving solver → sagittal abdominal diameter (SAD; not observable from a frontal photograph, solved from conservation of trunk volume under assumed values of the trunk mass fraction, density, length, and shape) → Ramanujan elliptical-perimeter computation on the section (TAD, SAD) → virtual three-dimensional waist circumference (C3).

The virtual three-dimensional waist circumference (C3) was computed under the assumption that the maximum-girth abdominal cross-section approximates an ellipse with semi-axes a = TAD/2 and b = SAD/2, using Ramanujan’s second elliptical-perimeter approximation (Ramanujan, 1914). We refer to C3 as a virtual circumference throughout to make explicit that it is reconstructed from a photograph, recorded height, and recorded weight rather than measured directly. Because SAD is solved from body mass, C3 necessarily shares information with BMI; the photographic transverse diameter, the only component of C3 that derives from the image, was therefore also analysed on its own. No value of C3 was imputed.

Four classes of computational sensitivity analyses were applied to C3: (i) Ramanujan approximation accuracy *versus* the exact elliptic integral; (ii) super-ellipse shape deviation under varied Lamé exponents; (iii) photographic angle bias under camera rotation up to ±15°; and (iv) Monte Carlo propagation of ±1% to ±10% Gaussian error in the photographic diameter, with the sagittal diameter re-solved for each perturbed value. As a check of plausibility, TAD, SAD, and C3 were related to recorded body weight and BMI and compared across the highest rank attained.

Career-peak height and weight were converted to BMI; elite professional rank was defined as career attainment of yokozuna, ōzeki, sekiwake, or komusubi; age at retirement was computed as retirement year minus birth year; and birth year was used as a continuous covariate (birth era, dichotomised at 1950, was used for descriptive stratification and in the originally specified models).

### Outcomes

The primary outcome was all-cause mortality, ascertained from published biographical death records and Japan Sumo Association obituary archives. Follow-up was computed from retirement to date of death or to 31 December 2025 for those alive, with administrative censoring at the latter date, in order to eliminate immortal time bias inherent in career-entry-anchored designs (reaching the top division requires several years of survival in pre-elite competition). Cause of death was classified into seven categories (cardiovascular, cerebrovascular, metabolic-cascade, neoplasm, respiratory, infection, and other) using a standardised abstraction protocol applied to free-text obituary descriptions. Cause of death was documented for 191 of the 467 deaths; all-cause results are therefore reported as the principal findings. Because a cause-specific hazard analysis that censors deaths of undocumented cause is valid only if documentation is unrelated to the covariates, the proportion of deaths with a documented cause was first compared by elite rank and period of death. Causes of death before 1990 were not analysed, for two reasons: diagnostic practice and cause-of-death classification in the Meiji, Taishō, and pre-war Shōwa periods cannot be mapped reliably to present-day categories, and no cause was documented for any non-elite death before 1946. Cause-specific analysis was therefore restricted to follow-up from 1 January 1990, the only period in which a cause was documented for more than half of deaths in both rank groups; wrestlers who had retired earlier entered the risk set on that date (delayed entry). Cause-specific hazards were estimated for cardiometabolic death (cardiovascular, cerebrovascular, and metabolic-cascade categories combined) and, as a negative-control outcome, for neoplastic death, with the covariates of the primary model and birth year as a continuous covariate. Undocumented causes were multiply imputed (200 imputations) from a multinomial logistic model including elite rank, age at death, year of death, and BMI, with a bootstrap draw of the imputation model for each imputation, and estimates were combined using Rubin’s rules (Rubin, 1987); this assumes that, conditional on these variables, documentation was unrelated to the cause of death. The conventional analysis censoring deaths of undocumented cause is reported for comparison. A secondary analysis used the narrower outcome of cardiac death alone (the cardiovascular category). Because diagnostic technology and the recording of causes of death also changed after 1990, most notably with the introduction of ICD-10 and the revised death certificate in Japan in 1995, the primary cause-specific analysis was repeated with follow-up starting in 1995 and in 2000.

### Statistical analysis

We followed the Strengthening the Reporting of Observational Studies in Epidemiology (STROBE) statement (Von Elm et al., 2007). The analysis was designed as a prognostic-factor analysis: the models describe associations between career-period characteristics and post-retirement mortality, and coefficients are not interpreted as causal effects, because no information on body size, lifestyle, or morbidity after retirement was available. The primary analysis used the primary cohort (birth year <1970) with retirement as the time origin. Cox proportional-hazards models estimated hazard ratios (HRs) with 95% CIs, with the proportional-hazards assumption verified by Schoenfeld residuals. Covariates were selected *a priori* as the career-period characteristics available for all wrestlers: age at retirement, which fixes the age at entry into the risk set; birth year, to account for the secular decline in mortality; elite rank, as a marker of competitive load and status; and body size. Because C3 is reconstructed in part from body mass and is almost collinear with BMI (*r* = 0.95), the two were not treated as separable: models entered one body-size measure at a time—no measure (Model 0), C3 (Model A), BMI (Model B), and the photographic transverse diameter (Model T1)—and were compared by Harrell’s C; the models entering BMI together with C3 (Model C) or with the photographic diameter (Model T2) are reported to show how much prognostic information each adds to BMI, and mutually adjusted coefficients are not interpreted as total effects (Westreich & Greenland, 2013). The original submission adjusted for birth cohort with a dichotomous indicator (post-1950 birth). Because body size rose and mortality fell progressively across birth cohorts, a two-level indicator leaves residual confounding; birth year was therefore entered as a continuous covariate in all models, stratification of the baseline hazard by birth decade was used as a check, and results with the original indicator are reported alongside (Table S1). C3 was further examined in alternative parameterisations (log-transformed, quartile, C3-per-kilogram ratio, and restricted cubic spline) and through an Elite × C3 interaction term, and under alternative geometric assumptions of the solver.

Standardised mortality ratios (SMRs) were computed against age- and calendar-year-stratified Japanese male reference mortality rates derived from the IPSS Japan Mortality Database (1947–2024, ages 25–95; National Institute of Population and Social Security Research, 2024) with Gompertz parameter extrapolation (Gompertz, 1825) for pre-1947 periods anchored on documented historical Japanese demographic statistics (Ministry of Health, Labour and Welfare, 2022). Expected deaths were computed by per-wrestler numerical integration of the age × calendar-year mortality surface over the post-retirement follow-up interval, and 95% CIs for SMRs were derived by the Byar approximation. As sensitivity analyses, SMRs were also computed using a simplified single-rate reference (0.012 per person-year), and a career-entry-anchored Cox model was reported for direct comparison with the prior literature (Hoshi & Inaba, 1995; Kanda et al., 2009). As a further analysis, career length (retirement age minus age at entry into sumo) and age at entry into sumo were entered alongside career-peak BMI (primary cohort, *n* = 604) in a model using attained age as the time scale, with left truncation at the age of retirement, and adjusting for birth year as a continuous covariate; age at retirement was not entered because it equals entry age plus career length, so that on the retirement-anchored time scale career length would simply carry the age effect. Career length is a proxy for the period of exposure to an occupational environment demanding high body mass; it does not represent the duration of obesity, because the timing of obesity onset was not recorded. The birth year ≥1970 sensitivity subset was reserved for descriptive sensitivity analysis. All analyses were conducted using Python v3.12 with pandas, lifelines v0.27, scikit-learn v1.5, and scipy v1.13.

Analysis code for the retirement-anchored and cause-specific Cox models, the volume-conserving sagittal-diameter solver, the Ramanujan-based C3 computation, the SMR calculations with Byar confidence intervals, and the C3 sensitivity analyses was written with the assistance of a large language model (Anthropic Claude, Opus 4.7; Anthropic PBC; https://www.anthropic.com/claude) under the author’s direct supervision; all code and numerical output were verified by the author.

## Results

### Cohort characteristics

After exclusion of 124 wrestlers flagged outside the main analytical set and 87 wrestlers with missing values on any required variable, the analytical cohort comprised 767 of 978 wrestlers in the source cohort (Fig. S1). The primary cohort (birth year <1970), used as the primary analysis, comprised 604 wrestlers with 467 deaths over a median post-retirement follow-up of 31.1 years (interquartile range (IQR) 22.0–39.4), and the birth year ≥1970 sensitivity subset comprised 163 wrestlers with 8 deaths over a median post-retirement follow-up of 12.0 years (Table 1). Median career-peak BMI was 36.2 kg/m^2^ (IQR 32.0–40.9), median weight 113 kg (98–132), median height 177 cm (173–182), median age at retirement 32.0 years (30.0–35.0), and 259 (43%) of the primary-cohort wrestlers attained an elite professional rank (yokozuna, ōzeki, sekiwake, or komusubi). A height-calibrated photographic measurement was available for 587 primary-cohort wrestlers (450 deaths), who formed the C3 analysis set.

**Table 1:** Baseline characteristics of the analytical cohort, by birth era.

**Characteristic**	**Primary cohort (birth year <1970)**	**Sensitivity cohort (birth year ≥1970)**	**Total cohort**
Wrestlers, n	604	163	767
Deaths, n	467	8	475
Median post-retirement follow-up, yr (IQR)	31.1 (22.0–39.4)	12.0 (6.0–16.0)	26.2 (14.4–37.0)
Person-years	18,579	1,881	20,460
Age at retirement, yr, median (IQR)	32.0 (30.0–35.0)	33.0 (30.0–36.0)	32.0 (30.0–35.0)
Career-peak height, cm, median (IQR)	177 (173–182)	185 (181–188)	179 (174–184)
Career-peak weight, kg, median (IQR)	113 (98–132)	154 (141–167)	120 (101–146)
BMI, kg/m^2^, median (IQR)	36.2 (32.0–40.9)	45.1 (41.7–49.9)	38.1 (32.7–43.8)
Wrestlers with a height-calibrated photograph (C3 analysis set), n	587	150	737
Deaths in the C3 analysis set, n	450	8	458
TAD (height-calibrated), cm, median (IQR)	44.9 (40.4–49.6)	50.7 (47.1–54.6)	46.3 (41.5–50.9)
SAD (volume-conserving solver), cm, median (IQR)	40.6 (36.4–45.9)	46.8 (41.8–51.6)	41.8 (37.1–46.9)
C3 virtual circumference, cm, median (IQR)	136.1 (126.8–145.4)	154.5 (148.9–160.9)	139.6 (129.2–150.9)
Elite professional rank, n (%)	259 (43)	71 (44)	330 (43)
Yokozuna	41 (7)	8 (5)	49 (6)
Ōzeki	39 (6)	14 (9)	53 (7)
Sekiwake	108 (18)	23 (14)	131 (17)
Komusubi	71 (12)	26 (16)	97 (13)
Pre-1950 birth, n (%)	465 (77)	0	465 (61)
Post-1950 birth, n (%)	139 (23)	163 (100)	302 (39)

**Notes.**

Values are median (interquartile range) or n (%). BMI, body mass index; TAD, transverse abdominal diameter, obtained from the career-peak photograph calibrated against recorded standing height; SAD, sagittal abdominal diameter, not observable from a frontal photograph and solved from conservation of trunk volume (trunk mass fraction 0.55, trunk density 1.03 g/cm^3^, trunk length 0.288 × height, cross-sectional area at maximal girth 1.20 × mean trunk cross-sectional area); C3, virtual three-dimensional waist circumference, the perimeter of the ellipse with axes TAD and SAD (Ramanujan’s approximation). TAD, SAD and C3 are given for the C3 analysis set. Elite professional rank denotes career attainment of yokozuna, ōzeki, sekiwake, or komusubi. Follow-up is retirement-anchored. Values of TAD, SAD and C3 in the birth year ≥1970 cohort should not be compared directly with the primary cohort because of the change in photographic style in the late twentieth century.

### Body-shape pipeline diagnostics

In the C3 analysis set the height-calibrated transverse abdominal diameter had a median of 44.9 cm (IQR 40.4–49.6; range 32.4–68.9), about one quarter of standing height, the solved sagittal diameter a median of 40.6 cm (36.4–45.9), giving an elliptical section slightly wider than deep (median SAD/TAD 0.92), and C3 a median of 136 cm (127–145; range 110–182) (Table 1). The photographic diameter, which uses no information on body mass, correlated with recorded weight (*r* = 0.54) and BMI (*r* = 0.42). C3, which combines the photographic diameter with recorded mass and height, correlated closely with BMI (*r* = 0.95; Fig. 2). All three measures rose with the highest rank attained, from maegashira to yokozuna (median TAD 43.6 to 48.9 cm, SAD 39.4 to 43.8 cm, C3 133 to 145 cm; Spearman *ρ* = 0.15, 0.22, and 0.29, all *p* < 0.001), as did BMI (34.6 to 39.9 kg/m^2^; *ρ* = 0.28). These relations support the plausibility of the reconstruction but do not establish its accuracy against a reference standard such as magnetic resonance imaging or direct tape measurement.

**Figure 2:**
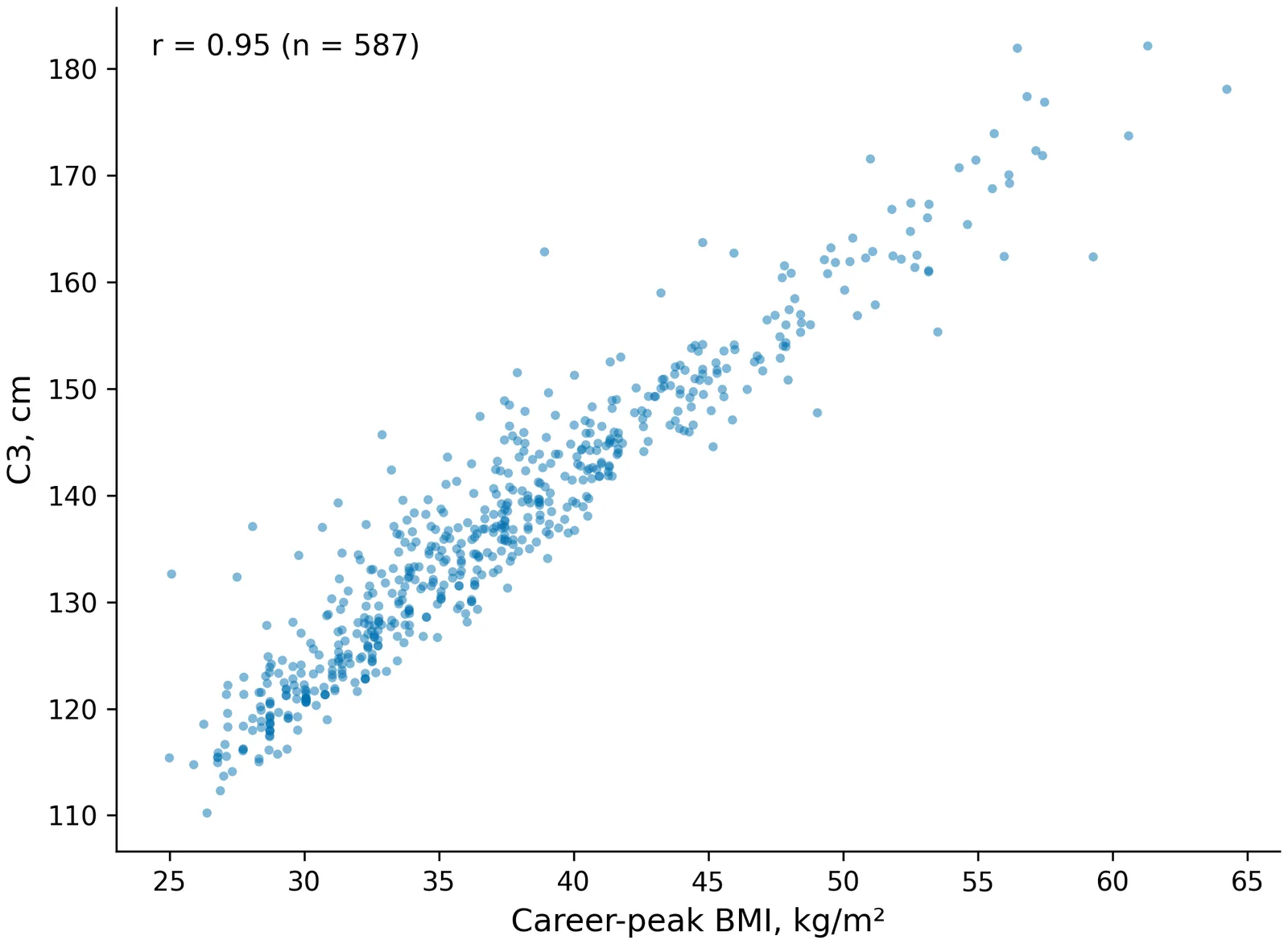
C3 and career-peak BMI. Scatter plot of the virtual three-dimensional waist circumference (C3) against body mass index (BMI) in the C3 analysis set (primary cohort with a height-calibrated photograph; *n* = 587). The close correlation (*r* = 0.95) reflects the reconstruction of the sagittal diameter from recorded body mass.

### Hazard ratios from retirement-anchored Cox regression

In retirement-anchored Cox regression adjusted for age at retirement, birth year, and elite rank, C3 was associated with all-cause mortality (Model A: HR 1.47 per standard deviation, 95% CI [1.29–1.69], *p* < 0.0001), as was career-peak BMI (Model B: HR 1.38, 1.22–1.55, *p* < 0.0001) (Fig. 3; Table 2). Harrell’s C was 0.656 for the model without a body-size measure, 0.675 with C3, and 0.672 with BMI. When the two almost collinear measures were entered together (Model C), discrimination was unchanged (0.674) and the coefficients could not be separated (C3 HR 1.62, 1.07–2.46; BMI HR 0.92, 0.63–1.33; variance inflation factor 10.7). The photographic transverse diameter, the only component of C3 that derives from the image, was not associated with mortality on its own (Model T1: HR 1.08, 0.95–1.22, *p* = 0.26; Harrell’s C 0.656) and added nothing to BMI (Model T2: HR 1.03, 0.90–1.17; Harrell’s C 0.672). The prognostic information in C3 is therefore that of recorded body mass and height. The association of C3 with mortality was graded, without evidence of non-linearity (highest *versus* lowest quartile HR 2.78, 1.92–4.04; restricted cubic spline, p for non-linearity 0.69), did not differ by rank (Elite × C3 interaction *p* = 0.68), and was insensitive to the geometric assumptions of the solver (HR 1.46 to 1.48 across assumptions under which median SAD ranged from 30.5 to 43.9 cm and median C3 from 121 to 141 cm; Table S2). In Model A, age at retirement (HR 1.04 per year, 1.01–1.07) and birth year (HR 0.76 per decade, 0.72–0.80) were associated with mortality and elite professional rank was not (HR 1.08, 0.88–1.33, *p* = 0.46); a career-entry-anchored model likewise showed no association with rank (HR 0.99, 0.81–1.21). Stratifying the baseline hazard by birth decade gave similar estimates (C3 HR 1.56, 1.35–1.79; BMI HR 1.45, 1.28–1.65). With the originally specified dichotomous era indicator, the single-measure estimates were attenuated (C3 HR 1.05, 0.93–1.18; BMI HR 1.11, 0.99–1.25) and the mutually adjusted coefficients of the collinear pair took opposite signs (C3 HR 0.58; BMI HR 1.82), a pattern that disappeared with continuous adjustment (Table S1); median career-peak BMI rose from 33.5 kg/m^2^ in the 1860–1879 birth cohorts to 44.8 kg/m^2^ in the 1960–1969 birth cohorts, and median C3 from 127 to 153 cm, while mortality declined. In the further model entering career length (years from entry into sumo to retirement) and age at entry into sumo alongside career-peak BMI, on the age time scale with continuous birth-year adjustment, career length was not associated with mortality (HR 0.98 per year, 95% CI [0.96–1.01], *p* = 0.21), later entry was associated with slightly lower mortality (HR 0.96 per year, 0.92–1.00, *p* = 0.049), and BMI per standard deviation was positively associated (HR 1.39, 1.24–1.56, *p* < 0.0001).

**Figure 3:**
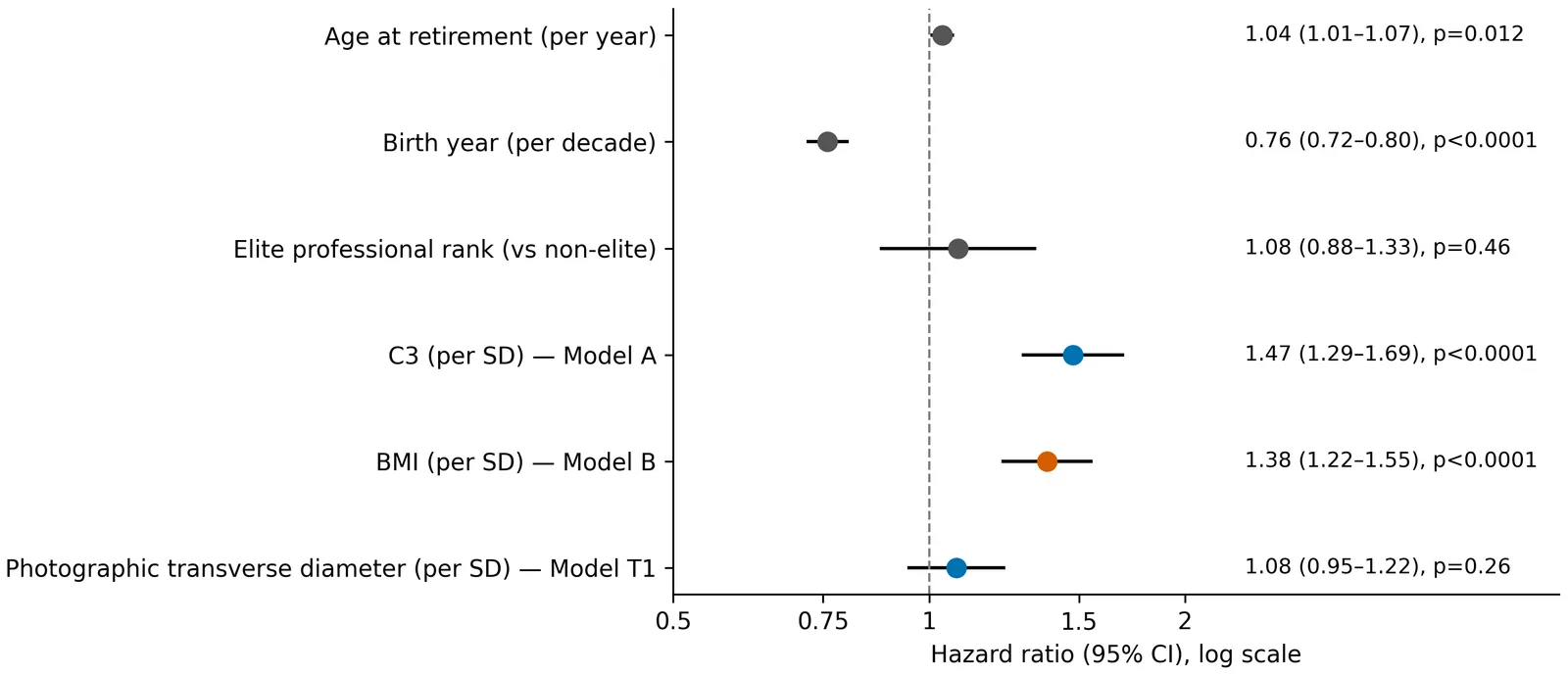
Hazard ratios for all-cause mortality from retirement-anchored Cox models entering one body-size measure at a time, C3 analysis set (*n* = 587, 450 deaths). Age at retirement, birth year, and elite professional rank are from Model A; C3 per SD from Model A, BMI per SD from Model B, and the photographic transverse diameter per SD from Model T1. All models are adjusted for age at retirement, birth year as a continuous covariate, and elite rank. The dashed line indicates HR = 1.0.

**Table 2:** Prognostic models for all-cause mortality after retirement entering one body-size measure at a time, with Harrell’s C; retirement-anchored Cox regression, C3 analysis set (primary cohort with a height-calibrated photograph; *n* = 587, deaths = 450).

	**Model 0: none**	**Model A: C3**	**Model B: BMI**	**Model C: BMI and C3**	**Model T1: TAD**	**Model T2: TAD and BMI**
Elite rank (*vs* non-elite)	1.24 (1.01–1.51)	1.08 (0.88–1.33)	1.11 (0.90–1.36)	1.08 (0.88–1.33)	1.22 (1.00–1.49)	1.10 (0.90–1.36)
Age at retirement (per year)	1.05 (1.02–1.08)	1.04 (1.01–1.07)	1.04 (1.01–1.07)	1.04 (1.01–1.06)	1.05 (1.02–1.08)	1.04 (1.01–1.07)
Birth year (per decade)	0.84 (0.80–0.87)	0.76 (0.72–0.80)	0.78 (0.75–0.82)	0.76 (0.71–0.80)	0.82 (0.78–0.86)	0.78 (0.74–0.82)
C3 (per SD)	–	1.47 (1.29–1.69)	–	1.62 (1.07–2.46)	–	–
BMI (per SD)	–	–	1.38 (1.22–1.55)	0.92 (0.63–1.33)	–	1.37 (1.21–1.55)
Photographic TAD (per SD)	–	–	–	–	1.08 (0.95–1.22)	1.03 (0.90–1.17)
Harrell’s C	0.656	0.675	0.672	0.674	0.656	0.672
Partial AIC	4,831.3	4,803.2	4,807.9	4,805.0	4,832.0	4,809.7

**Notes.**

Values are hazard ratios (95% confidence intervals). All models include elite rank, age at retirement, and birth year as a continuous covariate. C3 and BMI are almost collinear (*r* = 0.95; variance inflation factor 10.7), so the coefficients of Model C cannot be separated and are shown only for completeness; inference rests on Models A and B and on the comparison of discrimination. TAD is the only component of C3 that derives from the photograph. Results with the originally specified dichotomous era indicator and with stratification by birth decade are given in Supplemental Table S1.

BMI body mass index C3 virtual three-dimensional waist circumference TAD height-calibrated photographic transverse abdominal diameter SD standard deviation AIC Akaike information criterion — not included in the model

### Kaplan–Meier survival and standardised mortality ratios

Kaplan–Meier survival functions stratified by professional rank showed a modest separation between elite-ranked and non-elite wrestlers, while stratification by birth era demonstrated marked separation between pre-1950 and post-1950 births within the primary cohort (birth year <1970) (Fig. 4). Against age- and calendar-year-matched Japanese male reference rates derived from the IPSS Japan Mortality Database, standardised mortality ratios were 1.89 (95% CI [1.72–2.07]) for the primary cohort (birth year <1970) overall, 2.18 (95% CI [1.89–2.51]) for elite-ranked wrestlers, 1.72 (95% CI [1.52–1.94]) for non-elite wrestlers, 1.84 (95% CI [1.67–2.02]) for pre-1950 births, 2.57 (95% CI [1.85–3.48]) for post-1950 births, and 2.90 (95% CI [1.25–5.71]) for the birth year ≥1970 sensitivity subset (Fig. 5; Table S3). The full analytical cohort yielded an overall SMR of 1.90 (95% CI [1.73–2.08]). The simplified single-rate sensitivity analysis (reference 0.012 per person-year) showed substantially different point estimates only for the post-1950 and birth year ≥1970 strata (0.84 and 0.35, respectively), reflecting failure of the single-rate reference to account for the secular decline in Japanese male mortality.

**Figure 4:**
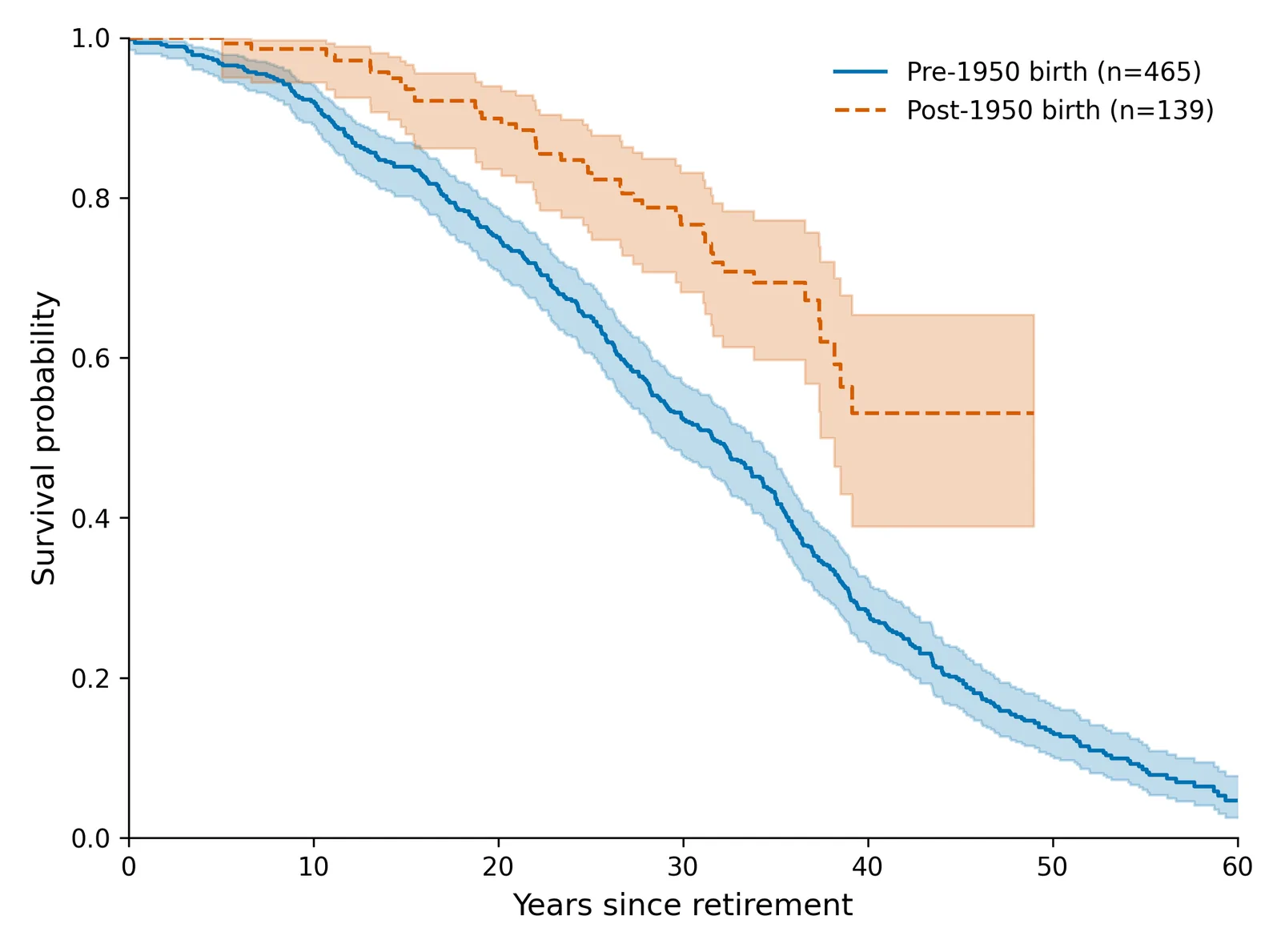
Kaplan–Meier survival by birth era, primary cohort (birth year <1970; *n* = 604, 467 deaths). Survival curves stratified by birth era (pre-1950 *vs* post-1950 within the primary cohort). Shaded regions indicate 95% confidence intervals. The relative within-cohort improvement contrasts with the era-independent absolute-rate excess in Fig. 5, reflecting that post-1950 wrestlers die less rapidly than pre-1950 wrestlers but still substantially faster than age × calendar-year-matched general-population Japanese males.

**Figure 5:**
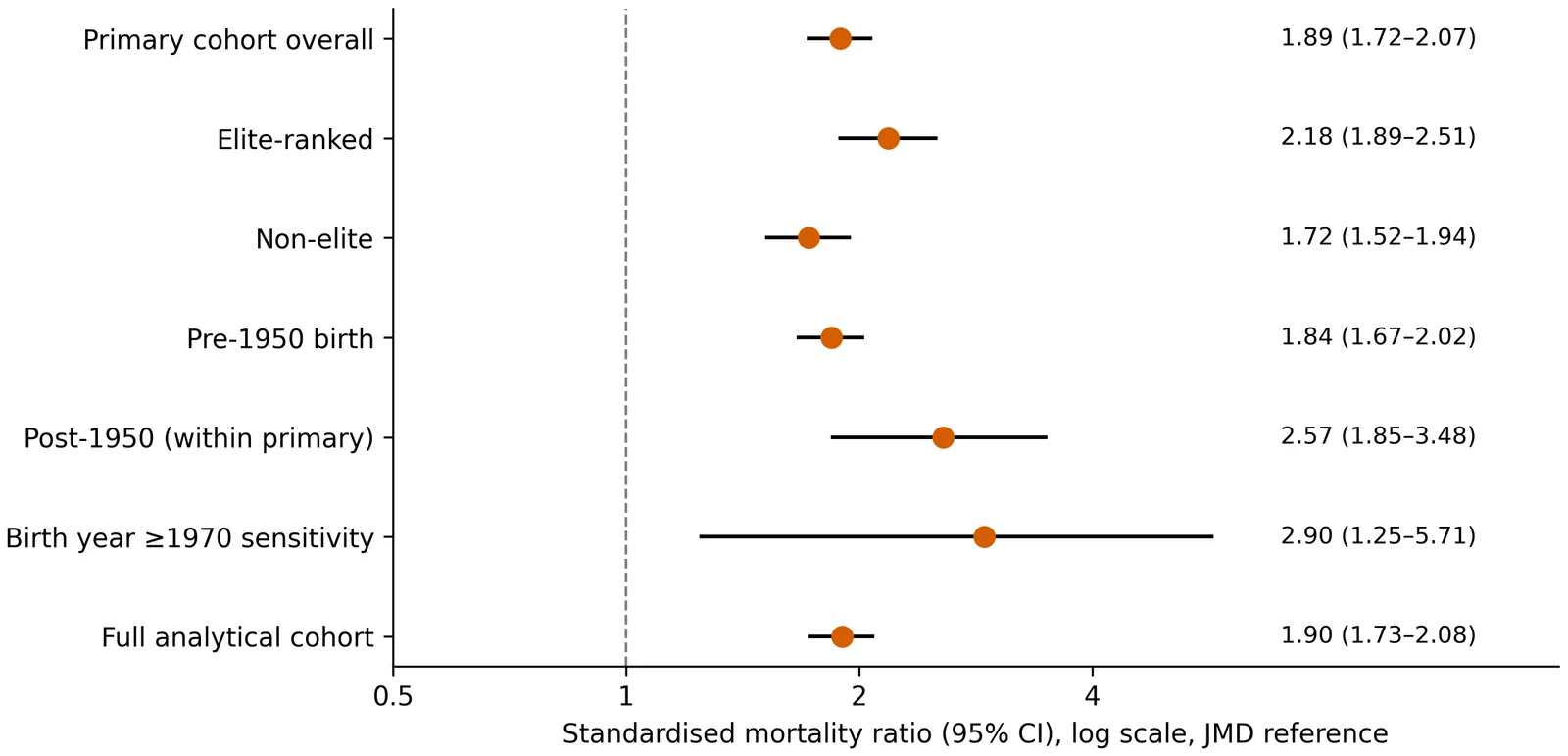
Standardised mortality ratios by sub-cohort, primary cohort (birth year <1970; *n* = 604, 467 deaths). Forest plot of age × calendar-year-stratified standardised mortality ratios (SMRs) computed against Japanese male reference rates from the IPSS Japan Mortality Database (1947–2024). The approximately twofold mortality excess persists across all historical strata of sufficient size: primary cohort overall SMR 1.89 (95% CI [1.72–2.07]), elite-ranked 2.18 (1.89–2.51), non-elite 1.72 (1.52–1.94), pre-1950 birth 1.84 (1.67–2.02), post-1950 birth 2.57 (1.85–3.48), and birth year ≥ 1970 sensitivity subset 2.90 (1.25–5.71); the bottom row shows the full analytical cohort (*n* = 767), 1.90 (1.73–2.08). The dashed line indicates SMR =1.0 (expected mortality). JMD, Japan Mortality Database.

### Cause-specific mortality

Cause of death was documented for 191 of the 467 deaths (41%), and documentation depended on both period and rank (Table S4). No cause was documented for any of the 43 non-elite deaths occurring before 1946, against 13 of 33 elite deaths, and a cause was documented for more than half of deaths in both rank groups only from 1990 onward (non-elite 46 of 81, 57%; elite 69 of 75, 92%); overall, a cause was recorded for 67% of elite and 21% of non-elite deaths. When deaths of undocumented cause were censored across the whole follow-up, elite rank was associated with death from any documented cause (HR 2.97, 95% CI [2.12–4.17]), which shows that such an analysis measures the completeness of documentation rather than cause-specific risk. In the analysis restricted to follow-up from 1990 (288 wrestlers; 151 deaths, of which 112 had a documented cause—51 cardiometabolic, 33 neoplastic, 28 other—and 39 were imputed), elite rank was associated with neither cardiometabolic death (HR 0.94, 95% CI [0.54–1.66]) nor neoplastic death (HR 1.12, 0.54–2.31) (Table 3). Career-peak BMI per standard deviation was associated with cardiometabolic death (HR 1.42, 1.08–1.87) but not with neoplastic death (HR 1.07, 0.73–1.56), and the same pattern was seen for C3 entered in its place (cardiometabolic HR 1.50, 1.08–2.07; neoplastic HR 1.20, 0.77–1.86). Results were similar for cardiac death alone (28 documented deaths; BMI HR 1.52, 1.04–2.22; C3 HR 1.67, 1.07–2.60; elite rank HR 0.69, 0.31–1.51). The findings were unchanged when follow-up started in 1995 (BMI and cardiometabolic death HR 1.44, 1.09–1.91; BMI and neoplastic death HR 1.09, 0.73–1.64; elite rank and cardiometabolic death HR 1.08, 0.60–1.93) or in 2000 (HR 1.58, 1.15–2.17; HR 1.11, 0.73–1.69; and HR 0.95, 0.49–1.83, respectively). When deaths of undocumented cause were instead censored, the elite-rank estimates were higher for both outcomes (cardiometabolic HR 1.42, 0.77–2.59; neoplastic HR 1.59, 0.74–3.39), consistent with the more complete documentation of elite deaths.

**Table 3:** Cause-specific hazard ratios for cardiometabolic and neoplastic death in follow-up from 1990, C3 analysis set (*n* = 288, deaths = 151), with multiple imputation of undocumented causes and, for comparison, with deaths of undocumented cause censored.

	**Multiple imputation (200 imputations)**		**Undocumented causes censored**	
	**Cardiometabolic**	**Neoplastic**	**Cardiometabolic**	**Neoplastic**
***Model with BMI***				
BMI (per SD)	1.42 (1.08–1.87)	1.07 (0.73–1.56)	1.52 (1.14–2.03)	1.11 (0.75–1.65)
Elite rank (*vs* non-elite)	0.94 (0.54–1.66)	1.12 (0.54–2.31)	1.42 (0.77–2.59)	1.59 (0.74–3.39)
***Model with C3***				
C3 (per SD)	1.50 (1.08–2.07)	1.20 (0.77–1.86)	1.63 (1.16–2.29)	1.28 (0.82–2.00)
Elite rank (*vs* non-elite)	0.93 (0.53–1.62)	1.11 (0.54–2.27)	1.42 (0.78–2.60)	1.54 (0.72–3.29)
Events (model with BMI)	70.2	43.6	51	33

**Notes.**

Values are hazard ratios (95% confidence intervals) from Cox models with follow-up beginning on 1 January 1990 (delayed entry for wrestlers who retired earlier), adjusted for elite rank, age at retirement, and birth year. Because BMI and C3 are almost collinear, each was entered in a separate model. Of the 151 deaths, 112 had a documented cause (51 cardiometabolic, 33 neoplastic, 28 other) and 39 were imputed from a multinomial logistic model including elite rank, age at death, year of death, and BMI; estimates from 200 imputations were combined using Rubin’s rules, and the number of events is the mean across imputations. Cardiometabolic death combines the cardiovascular, cerebrovascular, and metabolic-cascade categories; neoplastic death is a negative-control outcome. For cardiac death alone (28 documented deaths) the multiply imputed hazard ratios were 1.52 (1.04–2.22) for BMI and 1.67 (1.07–2.60) for C3. With follow-up starting in 1995 and in 2000, the hazard ratios for cardiometabolic death were 1.44 (1.09–1.91) and 1.58 (1.15–2.17) for BMI, and 1.54 (1.10–2.16) and 1.75 (1.21–2.55) for C3.

BMI body mass index C3 virtual three-dimensional waist circumference SD standard deviation

### Computational sensitivity analyses

Computational sensitivity analyses indicated that the C3 computation was numerically stable under the perturbations examined (Fig. S2). Ramanujan’s approximation deviated from the exact elliptic integral by less than 0.001% across the observed (TAD, SAD) range; a super-ellipse with Lamé exponent 2.2 instead of 2 increased C3 by 2.5 cm (1.8%); camera rotation about the vertical axis changed C3 by −0.04 cm at ±5° and by −0.35 cm (−0.3%) at ±15°, because a narrower apparent transverse diameter is offset by a deeper solved sagittal diameter; and ±5% Gaussian error in the photographic diameter propagated to a 0.8% relative standard deviation in C3 at the per-wrestler level. C3 is thus insensitive to error in the photograph, which is the counterpart of the finding that its variance is dominated by recorded mass and height. These analyses address computational stability, not validity against measured waist circumference.

## Discussion

This retrospective cohort study of 978 Japanese professional sumo wrestlers from the 1865–1990 birth cohort showed that top-division wrestlers experienced approximately twofold all-cause mortality compared with age- and calendar-year-matched Japanese males, with this excess persisting at approximately twofold across all historical strata of sufficient size for stable estimation (estimates in the small birth year ≥1970 sensitivity subset were imprecise), rather than improving in line with the secular decline in Japanese male mortality. With follow-up anchored at retirement to remove immortal time bias, career-peak body size was positively associated with long-term mortality once birth cohort was adjusted as a continuous variable, whether expressed as BMI (HR 1.38 per standard deviation) or as the reconstructed waist circumference C3 (HR 1.47); career length was not associated with mortality, and the coefficient for age at retirement reflects older age at entry into the post-retirement risk set rather than career length. Elite professional rank showed no independent all-cause association whether follow-up was anchored at retirement or, as in earlier analyses of sumo wrestlers (Hoshi & Inaba, 1995; Kanda et al., 2009), at career entry, and the reconstructed waist circumference showed discrimination similar to that of BMI but no clear incremental prognostic information, because the photographic measurement on which it rests added none to recorded body mass. Although the post-1950 birth stratum showed a modest within-cohort improvement, this improvement is referenced against the pre-1950 wrestlers of the same cohort, whose mortality was already extreme; against the contemporary general population, post-1950 and birth year ≥1970 wrestlers continue to die at approximately 2.5–2.9 times the expected rate.

Evidence from cohort studies of other weight-uncapped athletes provides directional, though not span-equivalent, support for these findings. The NIOSH American football cohort has documented two- to three-fold excess cardiovascular and neurodegenerative mortality concentrated in weight-uncapped positions over a 60-year span (Baron et al., 2012; Lehman et al., 2012; Nguyen et al., 2019; Venkataramani, Gandhavadi & Jena, 2018), the Finnish heavyweight weightlifter cohort has shown elevated cardiovascular event rates over a 45-year span (Sarna et al., 1993; Kujala et al., 1996; Pärssinen et al., 2000), and a 26-year contemporary professional wrestling cohort has reported markedly elevated cardiovascular mortality (Herman et al., 2014). The Japanese professional sumo record presented here exceeds the century-long mortality follow-up reported for French Olympic rowers (Antero-Jacquemin et al., 2014), and our finding that the mortality excess has not measurably attenuated over the documented period extends, rather than contradicts, this comparative evidence by suggesting that the mortality excess associated with this occupation has not been substantively offset by contemporary medical, nutritional, or post-career environmental improvements.

Our findings are consistent with and methodologically extend previous observational evidence on Japanese sumo wrestler mortality. Earlier obituary-based summaries and small cross-sectional series had reported overall mortality excess in the range of 1.5–2.5-fold but had used career-entry as the follow-up origin, which introduces immortal time bias because reaching the top division requires several years of survival in pre-elite competition; the largest prior published study covered 430 wrestlers entering the top division between 1926 and 1989 and could not separate elite-rank, era, and anthropometric associations with risk (Hoshi & Inaba, 1995; Kanda et al., 2009; Nishizawa et al., 1976). Our retirement-anchored estimates fall within the previously reported range against an age- and calendar-year-stratified reference, and avoid by design the immortal time that a career-entry origin introduces; in our data the elite-rank estimate was close to unity under either time origin (HR 1.08 retirement-anchored; HR 0.99 career-entry-anchored). In adjusted models, career-peak BMI was positively associated with long-term mortality, whereas career length was not; later age at entry showed only a weak inverse association with mortality. The coefficient for age at retirement reflects older age at entry into the post-retirement risk set—wrestlers who retire older begin follow-up at an older age—rather than the length of the career.

The era-independent persistence of the twofold mortality excess is biologically plausible. Career-induced obesity entails chronic positive energy balance, visceral adiposity, insulin resistance, hypertension, and accelerated cardiovascular ageing (Pi-Sunyer, 2009; Lavie, Milani & Ventura, 2009; Kivimäki et al., 2022; Nyberg et al., 2026), compounded by repeated musculoskeletal injury during competition and post-competition lifestyle dysregulation; these mechanisms operate continuously through the career and into retirement, and there is no *a priori* reason that contemporary medical care alone—delivered after symptoms emerge in retirement—should attenuate the underlying occupational exposure. Among the career-period measures, career-peak BMI was associated with mortality whereas career length was not. In models that included career-peak BMI, career length and the age of entry into sumo showed no positive association with mortality (career length HR 0.98, *p* = 0.21; entry age HR 0.96, *p* = 0.049), while BMI per standard deviation was positively associated (HR 1.39, *p* < 0.0001). This finding must be interpreted narrowly. Career length measures the period of exposure to an occupational environment demanding high body mass; it does not measure the duration of obesity, because career entry does not mark the onset of obesity and the timing of onset was not recorded. Nor was body size after retirement available. The absence of an association with career length should therefore not be interpreted as evidence that the duration of obesity is unrelated to mortality; reconstruction of individual weight trajectories from serial records would be required to address that question. With that caveat, the data point to body anthropometrics at career peak, rather than career timing, as the more informative career-period marker of later risk.

The reconstructed waist circumference requires careful interpretation. In the original analyses, C3 was reported to be orthogonal to BMI and unrelated to mortality. On subsequent examination, the abdominal diameters used in those analyses were found not to be on the height-calibrated scale described in the Methods, and their lack of correlation with body weight reflected that error rather than independent shape information. Recomputed from the height-calibrated photographic width, C3 takes values plausible for these athletes, rises with recorded weight, BMI, and competitive rank, and is associated with mortality in a graded manner with no sign of a ceiling within the range observed. Its association with mortality is as strong as that of BMI, but the two cannot be separated: the sagittal diameter is solved from body mass, so that most of the variance of C3 is that of mass and height, and the one component that comes from the photograph, the transverse diameter, neither predicted mortality nor improved discrimination beyond BMI. What this study can conclude is therefore limited. Historical portraits, combined with recorded height and weight, yield an anatomically coherent reconstruction of abdominal size, but in this cohort the image contributed no prognostic information beyond that already carried by recorded body mass. Whether abdominal shape or fat distribution carries such information cannot be determined from a frontal photograph, for the reasons given under Limitations.

An apparent exception merits comment. Elite-ranked wrestlers showed a higher absolute-rate SMR (2.18 *vs* 1.72) but no independent association in retirement-anchored Cox regression (HR 1.08, 95% CI [0.88–1.33], *p* = 0.46). The divergence reflects that elite-ranked wrestlers were larger—without a body-size measure the hazard ratio for elite rank was 1.24 (1.01–1.51; Table 2, Model 0)—and that age at retirement is consistently higher among elite-ranked wrestlers, who typically compete longer to defend their rank, so absolute-rate decomposition captures their excess deaths while the hazard-ratio analysis, which adjusts for age at retirement and body size, does not attribute independent all-cause risk to rank. Functionally these are consistent rather than contradictory readings of the same data. Whether the higher absolute mortality of elite-ranked wrestlers is concentrated in particular causes could be examined only for the recent period. Cause of death was documented far more often for elite than for non-elite wrestlers in every period, presumably because the deaths of prominent wrestlers were reported in more detail, and for deaths before the Second World War neither the documentation nor the diagnostic categories of the time support cause-specific inference. In follow-up from 1990, with undocumented causes multiply imputed, elite rank was not associated with cardiometabolic death; the apparent excess seen when undocumented deaths were censored reflects documentation rather than risk. Career-peak BMI, by contrast, was associated with cardiometabolic but not neoplastic death, a cause-specific pattern that parallels the position-dependent cardiovascular mortality of retired American football linemen (Baron et al., 2012; Miller et al., 2008) and the predominantly cardiovascular premature mortality of competitive powerlifters (Pärssinen et al., 2000), and that supports a cardiometabolic interpretation of the association between BMI and mortality. These estimates rest on 51 documented cardiometabolic deaths and on the assumption that documentation was unrelated to cause given rank, age, year of death, and BMI; confirmation will require cause-of-death ascertainment that is complete and independent of prominence, for example through linkage to death certificates.

The public health implications of these findings reframe the problem from a historical curiosity to an ongoing occupational-health concern. Despite the documented twofold mortality excess persisting across more than a century of professional sumo, we found no standardised post-retirement care framework specifically designed for heavyweight athletes in weight-uncapped elite disciplines—covering de-escalation of body mass, cardiometabolic surveillance, musculoskeletal rehabilitation, or psychological transition—and comparable framework gaps appear to exist for other weight-uncapped athletic populations. Our dataset and methodology provide an empirical platform on which such a framework can be designed: because career-peak body size, rather than career length, was associated with long-term mortality, and specifically with cardiometabolic death in the recent period, career-peak BMI may serve as a practical marker for identifying wrestlers who warrant intensified post-career cardiometabolic surveillance; whether reducing body mass after retirement lowers that risk cannot be inferred from these data. The higher standardised mortality ratio of elite-ranked wrestlers (2.18 *versus* 1.72) suggests that they, too, merit particular attention in post-career surveillance. Individual institutions in other weight-uncapped sports have run post-career programmes for body-mass de-escalation and life transition—for example, a university-based programme of nutritional support for graduating American football players not entering the professional leagues (Dohrmann, 2009)—but these are local initiatives rather than a standardised framework, and none has been evaluated for long-term cardiometabolic outcome.

### Limitations

Several limitations should be noted. First, our study relied on biographical records, so we cannot fully verify the accuracy of historical anthropometric measurements, although consistency between cohorts and the biological plausibility of the distributions support data quality; cause of death was documented for fewer than half of deaths, was particularly sparse in the earliest cohorts, and was recorded three times as often for elite-ranked as for non-elite wrestlers, and pre-war diagnostic labels could not be mapped reliably to present-day categories. Cause-specific analysis was therefore confined to follow-up from 1990 and relied on multiple imputation under a missing-at-random assumption that cannot be verified, with only 51 documented cardiometabolic deaths, so the cause-specific estimates should be regarded as supportive rather than definitive. Diagnostic technology and the recording of causes of death continued to change within this period, notably with the introduction of ICD-10 and the revised death certificate in 1995; the use of a composite outcome spanning the cardiovascular, cerebrovascular, and metabolic categories, adjustment for birth year, and the consistency of the estimates when follow-up started in 1995 or 2000 limit, but cannot exclude, the influence of such changes, and because causes were abstracted from obituaries and biographical sources rather than from death certificates, changes over time in how causes were described could not be examined. Second, the virtual three-dimensional waist circumference C3 represents a photograph-derived body-shape index rather than a directly measured anthropometric dimension: the transverse diameter is observable from a frontal photograph but the sagittal diameter is not, and although it is recovered under a physical volume constraint, it depends on assumed values for the trunk mass fraction and for the shape of the trunk, so C3 should be interpreted as a virtual circumference rather than a measured one. The absolute values of SAD and C3 vary with these assumptions (median C3 121 to 141 cm across those examined), whereas the hazard ratio per standard deviation does not. The photographic scale assumes that each standing portrait fills its frame; departures from this assumption, which may vary with the photographic conventions of each era, add error to TAD, and adjustment for birth year addresses only their systematic component. The finding that the photographic diameter added no prognostic information to BMI should not be over-interpreted as evidence that abdominal shape or visceral adiposity is unrelated to mortality in this population. C3 has not been validated against directly measured waist circumference or an imaging reference standard, because such measurements do not exist for the historical cohort; its correlations with recorded weight, BMI, and rank support plausibility, not accuracy. Non-differential error in the photographic diameter would attenuate any true association toward the null, so the absence of incremental prognostic information cannot be distinguished from insufficient measurement validity. Indeed, a photograph-derived external contour is dominated by subcutaneous fat and cannot resolve the visceral adiposity that most plausibly drives cardiometabolic death in this physique. Nor can it separate fat from lean tissue: sumo wrestlers have a very large fat-free mass, and BMI and percent body fat are discrepant in these athletes (Kondo et al., 1994; Hattori et al., 1999; Saito et al., 2003), so between-wrestler variation in trunk musculature is an additional source of error when C3, or BMI, is read as a marker of adiposity. This limitation cannot be remedied within a historical cohort: *in vivo* visceral-fat measurement by magnetic resonance imaging, computed tomography, or bioelectrical impedance cannot be applied retrospectively to wrestlers who are long deceased, so for historical populations the photograph is the only surviving source of body-shape information and the unmeasurable visceral component is irrecoverable. Directly testing whether shape-aware imaging adds prognostic information beyond BMI therefore cannot be done in this cohort and instead requires a prospective study of currently active or recently retired wrestlers with simultaneous image capture, tape-measured waist dimensions, and visceral-fat measurement; because no such paired dataset exists for sumo wrestlers, its prospective acquisition is a necessary next step. Third, our age- and calendar-year-stratified reference mortality rates relied on Gompertz extrapolation (Gompertz, 1825) for pre-1947 periods because the IPSS Japan Mortality Database begins in 1947; a fully empirical age × calendar-year SMR for pre-1947 cohorts will require either restriction to post-1947 deaths or the use of historical Japanese cohort life tables, and we present the simplified single-rate SMR as a transparent sensitivity analysis. Fourth, late-twentieth-century photographic-style change introduces a confound for birth year ≥1970 wrestlers, addressed by restricting the primary analysis to wrestlers born before 1970. Fifth, the birth year ≥1970 sensitivity subset (*n* = 163, eight deaths) is too small for reliable inference and is presented descriptively only.

Sixth, no information was available on body weight, waist size, diet, physical activity, smoking, alcohol consumption, or medical treatment after retirement. Body size may change substantially after retirement, and post-retirement trajectories may be more relevant to later mortality than career-peak values. Career-peak anthropometry should therefore be read as a marker of occupational exposure during the active career rather than of lifelong body size, and the associations reported here may be confounded or mediated by unmeasured post-retirement behavioural and clinical factors; this is the main reason the analysis is presented as prognostic rather than causal. Seventh, career length is not the duration of obesity, because the timing of obesity onset was not recorded; the null finding for career length therefore does not bear on whether the duration of obesity is related to mortality.

## Conclusions

Japanese professional sumo wrestlers from the 1865–1990 birth cohort experienced approximately twofold all-cause mortality compared with age- and calendar-year-matched general Japanese males, with this excess persisting at approximately twofold across historical strata of sufficient size. Among career-period measures, career-peak body size was positively associated with long-term mortality once birth cohort was adjusted continuously, whether expressed as BMI (HR 1.38 per standard deviation) or as the waist circumference C3 reconstructed from photographs, height, and weight (HR 1.47), whereas career length was not; the photographic component of C3 added no prognostic information to BMI; peak professional rank showed no independent all-cause association under either time origin and, in follow-up from 1990 with imputation of undocumented causes, no association with cardiometabolic death, whereas BMI and C3 were associated with cardiometabolic but not neoplastic death. Because career length does not measure the duration of obesity, the reconstruction has not been validated against measured waist circumference, and no post-retirement anthropometric or lifestyle data were available, the null findings for career length and for photographic shape should not be interpreted as evidence that obesity duration or abdominal fat distribution is unrelated to mortality. The era-independent persistence of the excess, together with the lack of a standardised post-retirement care framework specifically designed for heavyweight athletes in weight-uncapped elite disciplines, identifies an ongoing occupational-health concern. The dataset and the reusable AI-based body-shape reconstruction methodology presented here provide an empirical platform for the design of such a framework, and the analytical approach—retirement-anchored follow-up combined with age × calendar-year-stratified reference mortality and height-calibrated, volume-conserving reconstruction of body dimensions from photographic archives—is transferable to other historical occupational cohorts where direct anthropometric measurement is impossible.

##  Supplemental Information

10.7717/peerj.21924/supp-1 Supplemental Information 1 Individual-level analytic dataset (N=978 wrestlers reaching the makuuchi top division, 1865–1990 birth cohort) with Hepburn-romaji transliteration of all Japanese fields for non-Japanese-reading users Self-contained Python analysis pipeline for the anonymized English-language dataset accompanying the revised manuscript. The numbered scripts execute in order: 00_config.py defines paths, column-name constants, and analytical parameters, including the constants of the volume-conserving solver; 01_load_data.py applies the analytical cohort filter, computes the virtual three-dimensional waist circumference C3 from the height-calibrated photographic transverse diameter, a sagittal diameter solved from conservation of trunk volume, and Ramanujan’s elliptical-perimeter approximation, and reproduces the cohort counts and Table 1; 02_c3_pipeline.py verifies the photographic calibration and reports plausibility checks of TAD, SAD and C3 against recorded weight, BMI and rank (the Random Forest step of the original submission has been removed; no C3 value is imputed); 03_cox_regression.py runs retirement-anchored Cox proportional-hazards models entering one body-size measure at a time, under continuous birth-year adjustment and under the originally specified era indicator (Supplemental Table S1); 04_smr_analysis.py computes standardized mortality ratios against age x calendar-year-stratified Japanese male reference rates from the IPSS Japan Mortality Database with Byar 95% confidence intervals (Supplemental Table S3; requires the reference-rate file described in jmd_mortality_rates.md, which is hosted by a third party and not bundled); 05_sensitivity_c3.py runs the C3 computational sensitivity analyses and draws Supplemental Figure S2; 06_figures.py generates Figures 1-5 and Supplemental Figure S1 without titles inside the image files (when the Japan Mortality Database file is absent, Figure 5 is drawn from the SMR results saved in smr_results_reported.csv); 07_revision_models.py produces Table 2 (prognostic models with Harrell’s C), the geometric-assumption scenarios (Supplemental Table S2), the career-length model, and the cause-of-death ascertainment check; and 08_cause_specific_mi.py produces the cause-specific Cox models from 1990 with multiple imputation of undocumented causes (Table 3) and the documentation table (Supplemental Table S4). run_all.py executes all scripts; requirements.txt lists Python package versions; utils.py, README.md and data_dictionary.md provide shared helper functions, run instructions and variable definitions. The legacy diameter and C3 columns retained in the dataset are documented in data_dictionary.md and are not used in any analysis. The output/ folder contains the tables, figures and run log produced by a complete run, for comparison, and the codebook (Codebook_MASTER-SUMO.xlsx) is included.

10.7717/peerj.21924/supp-2 Supplemental Information 2 Python analysis scripts (revision R1) Description: Python scripts that reproduce the numerical results, tables and figures of the revised manuscript from the anonymized dataset: the cohort filter; computation of C3 from the height-calibrated photographic transverse diameter, a volume-conserving sagittal diameter and Ramanujan’s approximation; plausibility checks; retirement-anchored Cox models entering one body-size measure at a time, with Harrell’s C (Table 2; Supplemental Tables S1 and S2); the career-length model; cause-specific Cox models from 1990 with multiple imputation of undocumented causes (Table 3; Supplemental Table S4); computational sensitivity analyses of C3 (Supplemental Figure S2); and Figures 1–5 and Supplemental Figure S1. The age × calendar-year SMRs (Supplemental Table S3) additionally require the Japan Mortality Database reference rates, which are hosted by a third party; instructions are given in jmd_mortality_rates.md. See README.md for the run order and data_dictionary.md for variable definitions.

10.7717/peerj.21924/supp-3 Supplemental Information 3 STROBE checklist

10.7717/peerj.21924/supp-4 Supplemental Information 4 Participant flow Flow of wrestlers from the source cohort (*N* = 978) to the analytical cohort (*n* = 767), the primary cohort (birth year <1970; *n* = 604), the C3 analysis set (primary-cohort wrestlers with a height-calibrated photograph; *n* = 587), and the cause-specific analysis (under follow-up from 1 January 1990; *n* = 288). All counts are computed from the deposited dataset.

10.7717/peerj.21924/supp-5 Supplemental Information 5 Computational sensitivity of C3 to cross-sectional shape and camera rotation (A) Deviation of C3 from the elliptical value when the abdominal section is modelled as a super-ellipse with Lamé exponent n (*n* = 2 corresponds to an ellipse). (B) Difference between apparent and true C3 under rotation of the camera about the vertical axis, computed from the projected width of an elliptical section with the sagittal diameter re-solved from the apparent transverse diameter. Points are means and bars are standard deviations across wrestlers with a height-calibrated photograph (*n* = 737).

10.7717/peerj.21924/supp-6 Supplemental Information 6 Codebook for the anonymized analytic dataset Sheet “Codebook” gives the value-to-label mapping of all categorical variables; sheet “Continuous_variables” describes continuous and free-text variables, including the height-calibrated photographic measurements used to compute C3 and the legacy diameter and C3 columns that are retained for traceability but not used in any analysis; sheet “README” explains the structure.

10.7717/peerj.21924/supp-7 Supplemental Information 7 Hazard ratios for all-cause mortality from retirement-anchored Cox regression under alternative specifications, C3 analysis set (n=587, deaths=450) HR, hazard ratio; CI, confidence interval; SD, standard deviation; BMI, body mass index; C3, virtual three-dimensional waist circumference. Models A–C adjust for birth year as a continuous covariate; the “original era indicator” models use the dichotomous indicator (post-1950 birth) specified in the original submission. With the indicator, residual confounding by birth cohort attenuates the single-measure estimates, and the mutually adjusted coefficients of the almost collinear BMI and C3 take opposite signs; with continuous adjustment they do not. In a restricted cubic spline model for C3 (four knots) there was no evidence of non-linearity (likelihood-ratio *χ*^2^ = 0.74, 2 df, *p* = 0.69). All values are produced by the deposited code (03_cox_regression.py, 07_revision_models.py). This table replaces the former Table 2, which duplicated the forest plot.

10.7717/peerj.21924/supp-8 Supplemental Information 8 Sensitivity of the reconstructed sagittal diameter, of C3, and of the hazard ratio for C3 to the geometric assumptions of the volume-conserving solver, C3 analysis set (n=587, deaths=450) The general-population values are a trunk mass fraction of 0.497 and a trunk of uniform cross-section (Dempster, 1955; Drillis & Contini, 1966; Winter, 2009). Absolute values of SAD and C3 depend on the assumption; the hazard ratio per standard deviation of C3 and the discrimination of the model do not. SAD, sagittal abdominal diameter; TAD, transverse abdominal diameter; C3, virtual three-dimensional waist circumference; BMI, body mass index; HR, hazard ratio.

10.7717/peerj.21924/supp-9 Supplemental Information 9 Standardised mortality ratios (SMRs), by sub-cohort and reference rate * Primary SMR analysis: expected deaths computed by per-wrestler integration of age × calendar-year-stratified Japanese male reference mortality rates from the IPSS Japan Mortality Database (JMD, 1947–2024), with Gompertz extrapolation for pre-1947 periods. †Sensitivity analysis using a single reference rate of 0.012 per person-year, presented for transparency; the inversion of the post-1950 and birth year ≥1970 estimates illustrates how a single-rate reference fails to account for the secular decline in Japanese male mortality. 95% confidence intervals by the Byar approximation. This table contains the numerical results formerly presented as Table 3, which duplicated the forest plot (Figure 5).

10.7717/peerj.21924/supp-10 Supplemental Information 10 Documentation of cause of death by period of death and elite rank, primary cohort (467 deaths) Values are the number of deaths with a documented cause over the number of deaths (percentage). Elite rank: highest rank attained was yokozuna, ōzeki, sekiwake, or komusubi. No cause was documented for any non-elite death before 1946, and a cause was documented for more than half of deaths in both rank groups only from 1990 onward; cause-specific analysis was therefore restricted to follow-up from 1990.

## Additional Information and Declarations

### Data Availability

The following information was supplied regarding data availability: The data and code are available in the Supplemental Files.

## References

[ref-1] Antero-Jacquemin J, Desgorces FD, Dor F, Sedeaud A, Haida A, Le Van P, Toussaint JF (2014). Row for your life: a century of mortality follow-up of French Olympic rowers. PLOS ONE.

[ref-2] Baron SL, Hein MJ, Lehman EJ, Gersic CM (2012). Body mass index, playing position, race, and the cardiovascular mortality of retired professional football players. American Journal of Cardiology.

[ref-3] Dempster WT (1955). Space requirements of the seated operator: geometrical, kinematic, and mechanical aspects of the body with special reference to the limbs. WADC Technical Report 55-159.

[ref-4] Dohrmann G (2009). I want my body back. Sports Illustrated, 8 June 2009. https://www.si.com/more-sports/2009/06/03/shedding-pounds.

[ref-5] Drillis R, Contini R (1966). Body segment parameters. Report No. 1166-03.

[ref-6] GBD 2021 Adult BMI Collaborators (2025). Global, regional, and national prevalence of adult overweight and obesity, 1990–2021, with forecasts to 2050: a forecasting study for the Global Burden of Disease Study 2021. The Lancet.

[ref-7] Gompertz B (1825). On the nature of the function expressive of the law of human mortality, and on a new mode of determining the value of life contingencies. Philosophical Transactions of the Royal Society of London.

[ref-8] Hattori K, Kondo M, Abe T, Tanaka S, Fukunaga T (1999). Hierarchical differences in body composition of professional Sumo wrestlers. Annals of Human Biology.

[ref-9] Herman CW, Conlon ASC, Rubenfire M, Burghardt AR, McGregor SJ (2014). The very high premature mortality rate among active professional wrestlers is primarily due to cardiovascular disease. PLOS ONE.

[ref-10] Heshmati HM (2025). Lifestyle, comorbidities, and longevity of sumo wrestlers. Journal of the Endocrine Society.

[ref-11] Hoshi A, Inaba Y (1995). Risk factors for mortality and mortality rate of sumo wrestlers. Japanese Journal of Hygiene.

[ref-12] Kanda H, Hayakawa T, Tsuboi S, Mori Y, Takahashi T, Fukushima T (2009). Higher body mass index is a predictor of death among professional sumo wrestlers. Journal of Sports Science & Medicine.

[ref-13] Kanehisa H, Kondo M, Ikegawa S, Fukunaga T (1998). Body composition and isokinetic strength of professional sumo wrestlers. European Journal of Applied Physiology.

[ref-14] Kivimäki M, Strandberg T, Pentti J, Nyberg ST, Frank P, Jokela M, Ervasti J, Suominen SB, Vahtera J, Sipilä PN, Lindbohm JV, Ferrie JE (2022). Body-mass index and risk of obesity-related complex multimorbidity: an observational multicohort study. The Lancet Diabetes & Endocrinology.

[ref-15] Kondo M, Abe T, Ikegawa S, Kawakami Y, Fukunaga T (1994). Upper limit of fat-free mass in humans: a study on Japanese Sumo wrestlers. American Journal of Human Biology.

[ref-16] Kujala UM, Sarna S, Kaprio J, Koskenvuo M (1996). Hospital care in later life among former world-class Finnish athletes. Journal of the American Medical Association.

[ref-17] Lavie CJ, Milani RV, Ventura HO (2009). Obesity and cardiovascular disease: risk factor, paradox, and impact of weight loss. Journal of the American College of Cardiology.

[ref-18] Lehman EJ, Hein MJ, Baron SL, Gersic CM (2012). Neurodegenerative causes of death among retired National Football League players. Neurology.

[ref-19] Lemez S, Baker J (2015). Do elite athletes live longer? A systematic review of mortality and longevity in elite athletes. Sports Medicine - Open.

[ref-20] Miller MA, Croft LB, Belanger AR, Romero-Corral A, Somers VK, Roberts AJ, Goldman ME (2008). Prevalence of metabolic syndrome in retired National Football League players. American Journal of Cardiology.

[ref-21] Ministry of Health, Labour and Welfare (2022). The 23rd Complete Life Tables for Japan 2020. https://www.mhlw.go.jp/english/database/db-hw/lifetb23nd/dl/introduction.pdf.

[ref-22] National Institute of Population and Social Security Research (2024). Japan Mortality Database. https://www.ipss.go.jp/p-toukei/JMD/.

[ref-23] NCD Risk Factor Collaboration (2024). Worldwide trends in underweight and obesity from 1990 to 2022: a pooled analysis of 3663 population-representative studies with 222 million children, adolescents, and adults. The Lancet.

[ref-24] Nguyen VT, Zafonte RD, Chen JT, Kponee-Shovein KZ, Paganoni S, Pascual-Leone A, Speizer FE, Baggish AL, Taylor Jr HA, Nadler LM, Courtney TK, Connor A, Grashow R, Whittington AJ, Weisskopf MG (2019). Mortality among professional American-style football players and professional American baseball players. JAMA Network Open.

[ref-25] Nishizawa T, Akaoka I, Nishida Y, Kawaguchi Y, Hayashi E, Yoshimura T (1976). Some factors related to obesity in the Japanese sumo wrestler. American Journal of Clinical Nutrition.

[ref-26] Nyberg ST, Kivimäki M, Pentti J, Frank P, Jokela M, Ervasti J, Suominen SB, Vahtera J, Strandberg T (2026). Adult obesity and risk of severe infections: a multicohort study with global burden estimates. The Lancet.

[ref-27] Pärssinen M, Kujala U, Vartiainen E, Sarna S, Seppälä T (2000). Increased premature mortality of competitive powerlifters suspected to have used anabolic agents. International Journal of Sports Medicine.

[ref-28] Pi-Sunyer FX (2009). The medical risks of obesity. Postgraduate Medicine.

[ref-29] Ramanujan S (1914). Modular equations and approximations to *π*. Quarterly Journal of Pure and Applied Mathematics.

[ref-30] Rubin DB (1987). Multiple imputation for nonresponse in surveys.

[ref-31] Rubino F, Cummings DE, Eckel RH, Cohen RV, Wilding JPH, Brown WA, Stanford FC, Batterham RL, Farooqi IS, Farpour-Lambert NJ, Le Roux CW, Sattar N, Baur LA, Morrison KM, Misra A, Kadowaki T, Tham KW, Sumithran P, Garvey WT, Kirwan JP, Fernández-Real JM, Corkey BE, Toplak H, Kokkinos A, Kushner RF, Branca F, Valabhji J, Blüher M, Bornstein SR, Grill HJ, Ravussin E, Gregg E, Al Busaidi NB, Alfaris NF, Al Ozairi E, Carlsson LMS, Clément K, Després JP, Dixon JB, Galea G, Kaplan LM, Laferrère B, Laville M, Lim S, Luna Fuentes JR, Mooney VM, Nadglowski J, Urudinachi A, Olszanecka-Glinianowicz M, Pan A, Pattou F, Schauer PR, Tschöp MH, Vander Merwe MT, Vettor R, Mingrone G (2025). Definition and diagnostic criteria of clinical obesity: a Lancet Diabetes & Endocrinology Commission. The Lancet Diabetes & Endocrinology.

[ref-32] Runacres A, Mackintosh KA, McNarry MA (2021). Health consequences of an elite sporting career: long-term detriment or long-term gain? A meta-analysis of 165,000 former athletes. Sports Medicine.

[ref-33] Saito K, Nakaji S, Umeda T, Shimoyama T, Sugawara K, Yamamoto Y (2003). Development of predictive equations for body density of sumo wrestlers using B-mode ultrasound for the determination of subcutaneous fat thickness. British Journal of Sports Medicine.

[ref-34] Sarna S, Sahi T, Koskenvuo M, Kaprio J (1993). Increased life expectancy of world class male athletes. Medicine & Science in Sports & Exercise.

[ref-35] Venkataramani AS, Gandhavadi M, Jena AB (2018). Association between playing American football in the National Football League and long-term mortality. Journal of the American Medical Association.

[ref-36] Von Elm E, Altman DG, Egger M, Pocock SJ, Gøtzsche PC, Vandenbroucke JP (2007). The Strengthening the Reporting of Observational Studies in Epidemiology (STROBE) statement: guidelines for reporting observational studies. The Lancet.

[ref-37] Westreich D, Greenland S (2013). The Table 2 fallacy: presenting and interpreting confounder and modifier coefficients. American Journal of Epidemiology.

[ref-38] Winter DA (2009). Biomechanics and motor control of human movement.

