# Supplementary material for: Predictors of post-retirement mortality in athletes required to maintain very high body mass: a retrospective cohort study using AI-driven three-dimensional body reconstruction, 1865–1990
Source: PeerJ. 2026 Nov 10;14:e21924. doi: 10.7717/peerj.21924 (PMC13762328; doi:10.7717/peerj.21924)
Supplement: Supplementary file 2 [file peerj-14-21924-s003.docx]

**STROBE Statement—Checklist of items that should be included in reports of cohort studies**

*Manuscript (PeerJ #140524, revised version): “Predictors of post-retirement mortality in athletes required to maintain very high body mass: a retrospective cohort study using AI-driven three-dimensional body reconstruction, 1865–1990”*

| **Item** | **Topic** | **STROBE recommendation** | **Location in manuscript (line numbers refer to the line-numbered revised manuscript)** |
| --- | --- | --- | --- |
| 1 | **Title and abstract** | (a) Indicate the study's design with a commonly used term in the title or the abstract. (b) Provide in the abstract an informative and balanced summary of what was done and what was found. | (a) Title (L1–3: “a retrospective cohort study”) and Abstract – Methods (L17–25). (b) Abstract, L10–43. |
| 2 | **Background / rationale** | Explain the scientific background and rationale for the investigation being reported. | Introduction, L45–82. |
| 3 | **Objectives** | State specific objectives, including any prespecified hypotheses. | Introduction, L83–95 (aims; the analysis is stated to be prognostic and not to estimate causal effects, L92–95). |
| 4 | **Study design** | Present key elements of study design early in the paper. | Methods – Study design and participants, L98–102 (retrospective cohort study); Statistical analysis, L194–198 (prognostic-factor analysis; retirement as the time origin). |
| 5 | **Setting** | Describe the setting, locations, and relevant dates, including periods of recruitment, exposure, follow-up, and data collection. | Methods – Study design and participants, L98–112 (makuuchi division, Meiji to early Heisei eras; birth years 1865–1990); Outcomes, L161–166 (follow-up from retirement to death or 31 December 2025). |
| 6 | **Participants** | (a) Give the eligibility criteria, and the sources and methods of selection of participants. Describe methods of follow-up. (b) For matched studies, give matching criteria and number of exposed and unexposed. | (a) Methods – Study design and participants, L103–112 (eligibility, sources, exclusions, C3 analysis set); Outcomes, L161–166 (follow-up). (b) Not applicable (no matched design). |
| 7 | **Variables** | Clearly define all outcomes, exposures, predictors, potential confounders, and effect modifiers. Give diagnostic criteria, if applicable. | Methods – Procedures, L121–159 (TAD, SAD, C3, BMI, elite rank, age at retirement, birth year); Outcomes, L161–191 (all-cause mortality; cause-of-death categories; cardiometabolic, neoplastic and cardiac outcomes); Statistical analysis, L228–235 (career length, age at entry). |
| 8* | **Data sources / measurement** | For each variable of interest, give sources of data and details of methods of assessment (measurement). Describe comparability of assessment methods if there is more than one group. | Methods – Procedures, L121–147 (digitised portraits; MediaPipe Pose v0.10, YOLOv8; calibration against recorded standing height; volume-conserving solver; Ramanujan approximation); Outcomes, L161–168 (biographical death records, obituary archives; abstraction protocol); Statistical analysis, L218–222 (IPSS Japan Mortality Database reference rates). |
| 9 | **Bias** | Describe any efforts to address potential sources of bias. | Introduction L65–66 and Outcomes L162–166 (retirement-anchored follow-up to avoid immortal time bias); Outcomes L170–191 (differential documentation of cause of death by rank and period: restriction to follow-up from 1990, multiple imputation, negative-control outcome, 1995/2000 starts); Statistical analysis L203–214 (collinearity of C3 and BMI; continuous birth-year adjustment for residual confounding by birth cohort); Methods L107–108 (primary cohort restricted to birth year <1970 because of photographic-style change). |
| 10 | **Study size** | Explain how the study size was arrived at. | Methods – Study design and participants, L98–112 (full population of makuuchi wrestlers; source N=978 → analytical n=767 → primary n=604 → C3 analysis set n=587); Results, L246–256; Supplemental Figure S1. |
| 11 | **Quantitative variables** | Explain how quantitative variables were handled in the analyses. If applicable, describe which groupings were chosen and why. | Methods – Procedures L155–159 (BMI; birth year continuous; era dichotomised at 1950 for description); Statistical analysis L203–217 (C3, BMI and TAD per SD; quartiles, log transformation, C3-per-kilogram ratio, restricted cubic spline). |
| 12* | **Statistical methods** | (a) Describe all statistical methods, including those used to control for confounding. (b) Describe methods used to examine subgroups and interactions. (c) Explain how missing data were addressed. (d) If applicable, explain how loss to follow-up was addressed. (e) Describe any sensitivity analyses. | (a) Statistical analysis L193–237 (Cox proportional-hazards models entering one body-size measure at a time, adjusted for age at retirement, birth year and elite rank; Harrell’s C; SMRs against age × calendar-year reference rates); cause-specific hazards, Outcomes L176–191. (b) L215–217 (Elite × C3 interaction; alternative parameterisations); L228–235 (career length and age at entry on the attained-age time scale). (c) Methods L105–111 (exclusion of records missing required variables, n=87; C3 analysis set); Outcomes L182–186 (multiple imputation of undocumented causes of death, 200 imputations, Rubin’s rules). (d) Outcomes L162–164 (administrative censoring at 31 December 2025). (e) L148–154 (four classes of computational sensitivity analyses for C3); L186–191 (censoring analysis; 1995 and 2000 starts); L210–217 (original era indicator; stratification by birth decade; geometric assumptions of the solver); L225–227 (single-rate SMR; career-entry-anchored model). |
| 13* | **Participants** | (a) Report numbers of individuals at each stage. (b) Give reasons for non-participation at each stage. (c) Consider use of a flow diagram. | (a)–(b) Methods L105–112 and Results – Cohort characteristics, L246–256 (978 source → 124 flagged out → 87 missing → 767 analytical → 604 primary / 163 sensitivity → 587 C3 analysis set); Cause-specific mortality L325–327 (288 wrestlers under follow-up from 1990). (c) Flow diagram: Supplemental Figure S1. |
| 14* | **Descriptive data** | (a) Give characteristics of study participants and information on exposures and potential confounders. (b) Indicate number of participants with missing data for each variable of interest. (c) Summarise follow-up time. | (a) Results – Cohort characteristics, L246–256; Body-shape pipeline diagnostics, L258–269; Table 1. (b) Methods L105–111; Results L255–256 (height-calibrated photograph unavailable for 17 primary-cohort wrestlers); L317–322 (cause of death undocumented for 276 of 467 deaths; Supplemental Table S4). (c) Results L249–252 (median post-retirement follow-up 31.1 years; 18,579 person-years, Table 1). |
| 15* | **Outcome data** | Report numbers of outcome events or summary measures over time. | Results – Cohort characteristics L249–252 (467 deaths in the primary cohort; 8 in the sensitivity subset); Cause-specific mortality L317–328 (191 of 467 deaths with a documented cause; from 1990, 151 deaths: 51 cardiometabolic, 33 neoplastic, 28 other, 39 imputed); Table 1; Table 3. |
| 16 | **Main results** | (a) Give unadjusted and, if applicable, confounder-adjusted estimates and their precision; make clear which confounders were adjusted for and why. (b) Report category boundaries when continuous variables were categorized. (c) If relevant, translate relative risk into absolute risk for a meaningful time period. | (a) Results – Hazard ratios L271–301; Table 2 (Model 0 without a body-size measure and Models A–T2; adjustment set and its rationale in Methods L200–214); Figure 3; Supplemental Table S1. (b) C3 quartiles (L282–283). (c) Results – Kaplan–Meier survival and SMRs L303–315; Figures 4–5; Supplemental Table S3. |
| 17 | **Other analyses** | Report other analyses done – e.g. analyses of subgroups and interactions, and sensitivity analyses. | Results – Hazard ratios L281–301 (alternative C3 parameterisations; Elite × C3 interaction; geometric assumptions, Supplemental Table S2; original era indicator and birth-decade strata, Supplemental Table S1; career length and age at entry); Cause-specific mortality L317–339 (Table 3); Computational sensitivity analyses L341–351 (Supplemental Figure S2). |
| 18 | **Key results** | Summarise key results with reference to study objectives. | Discussion, L353–372; Conclusions, L549–572. |
| 19 | **Limitations** | Discuss limitations of the study, taking into account sources of potential bias or imprecision. Discuss both direction and magnitude of any potential bias. | Discussion – Limitations, L481–547 (seven numbered limitations, including the unvalidated status of C3, dependence of its absolute values on geometric assumptions, the missing-at-random assumption for cause of death, and the absence of post-retirement data; direction of bias from non-differential measurement error, L512–514). |
| 20 | **Interpretation** | Give a cautious overall interpretation considering objectives, limitations, multiplicity of analyses, results from similar studies, and other relevant evidence. | Discussion, L373–465 (comparison with other weight-uncapped athlete cohorts and earlier sumo studies; career length versus duration of obesity; interpretation of the reconstructed waist circumference; rank and cause-specific mortality). |
| 21 | **Generalisability** | Discuss the generalisability (external validity) of the study results. | Discussion L466–479 (public-health implications); Conclusions L566–572 (transferability to other historical occupational cohorts). |
| 22 | **Funding** | Give the source of funding and the role of the funders for the present study and, if applicable, for the original study on which the present article is based. | Declared in the submission system: “The authors received no funding for this work.” |

**Give such information separately for exposed and unexposed groups in cohort and case-control studies, and, if applicable, for cases and controls in case-control studies and for exposed and unexposed groups in cross-sectional studies.*

*Note: An Explanation and Elaboration article discusses each checklist item and gives methodological background and published examples of transparent reporting. The STROBE checklist is best used in conjunction with this article (freely available at https://www.strobe-statement.org).*
