# Supplementary material for: Predictors of post-retirement mortality in athletes required to maintain very high body mass: a retrospective cohort study using AI-driven three-dimensional body reconstruction, 1865–1990
Source: PeerJ. 2026 Nov 10;14:e21924. doi: 10.7717/peerj.21924 (PMC13762328; doi:10.7717/peerj.21924)
Supplement: Supplementary file 3 [file peerj-14-21924-s007.docx]

**Supplemental Table S1.** Hazard ratios for all-cause mortality from retirement-anchored Cox regression under alternative specifications, C3 analysis set (n=587, deaths=450).

| **Model and covariate** | **HR** | **95% CI** | **p-value** |
| --- | --- | --- | --- |
| ***Model A: C3, continuous birth year*** | | | |
| Age at retirement, per year | 1.04 | 1.01–1.07 | 0.012 |
| Birth year, per decade | 0.76 | 0.72–0.80 | <0.0001 |
| Elite professional rank (vs non-elite) | 1.08 | 0.88–1.33 | 0.46 |
| C3, per SD | 1.47 | 1.29–1.69 | <0.0001 |
| ***Model B: BMI, continuous birth year*** | | | |
| Age at retirement, per year | 1.04 | 1.01–1.07 | 0.004 |
| Birth year, per decade | 0.78 | 0.75–0.82 | <0.0001 |
| Elite professional rank (vs non-elite) | 1.11 | 0.90–1.36 | 0.33 |
| BMI, per SD | 1.38 | 1.22–1.55 | <0.0001 |
| ***Model C: BMI and C3, continuous birth year*** | | | |
| Age at retirement, per year | 1.04 | 1.01–1.06 | 0.014 |
| Birth year, per decade | 0.76 | 0.71–0.80 | <0.0001 |
| Elite professional rank (vs non-elite) | 1.08 | 0.88–1.33 | 0.47 |
| BMI, per SD | 0.92 | 0.63–1.33 | 0.65 |
| C3, per SD | 1.62 | 1.07–2.46 | 0.024 |
| ***Original era indicator: C3*** | | | |
| Age at retirement, per year | 1.09 | 1.06–1.12 | <0.0001 |
| Post-1950 birth (vs pre-1950) | 0.44 | 0.31–0.62 | <0.0001 |
| Elite professional rank (vs non-elite) | 1.08 | 0.87–1.32 | 0.49 |
| C3, per SD | 1.05 | 0.93–1.18 | 0.43 |
| ***Original era indicator: BMI*** | | | |
| Age at retirement, per year | 1.09 | 1.06–1.12 | <0.0001 |
| Post-1950 birth (vs pre-1950) | 0.42 | 0.29–0.59 | <0.0001 |
| Elite professional rank (vs non-elite) | 1.05 | 0.85–1.29 | 0.66 |
| BMI, per SD | 1.11 | 0.99–1.25 | 0.069 |
| ***Original era indicator: BMI and C3*** | | | |
| Age at retirement, per year | 1.09 | 1.06–1.11 | <0.0001 |
| Post-1950 birth (vs pre-1950) | 0.47 | 0.33–0.67 | <0.0001 |
| Elite professional rank (vs non-elite) | 1.09 | 0.89–1.34 | 0.42 |
| BMI, per SD | 1.82 | 1.26–2.63 | 0.001 |
| C3, per SD | 0.58 | 0.40–0.85 | 0.006 |
| ***C3 alternative parameterisations (as Model A)*** | | | |
| C3 log-transformed (per SD) | 1.48 | 1.29–1.69 | <0.0001 |
| C3 quartile Q4 vs Q1 | 2.78 | 1.92–4.03 | <0.0001 |
| C3-per-kg ratio (per SD) | 0.69 | 0.60–0.80 | <0.0001 |
| C3 x elite-rank interaction | 0.96 | 0.78–1.17 | 0.68 |
| ***Career-entry-anchored (C3, continuous birth year)*** | | | |
| Elite professional rank (vs non-elite) | 0.99 | 0.81–1.21 | 0.95 |
| Birth year, per decade | 0.75 | 0.72–0.79 | <0.0001 |
| C3, per SD | 1.42 | 1.25–1.62 | <0.0001 |
| ***Stratified by birth decade: C3*** | | | |
| C3, per SD | 1.56 | 1.35–1.79 | <0.0001 |
| ***Stratified by birth decade: BMI*** | | | |
| BMI, per SD | 1.45 | 1.28–1.65 | <0.0001 |

HR, hazard ratio; CI, confidence interval; SD, standard deviation; BMI, body mass index; C3, virtual three-dimensional waist circumference. Models A–C adjust for birth year as a continuous covariate; the “original era indicator” models use the dichotomous indicator (post-1950 birth) specified in the original submission. With the indicator, residual confounding by birth cohort attenuates the single-measure estimates, and the mutually adjusted coefficients of the almost collinear BMI and C3 take opposite signs; with continuous adjustment they do not. In a restricted cubic spline model for C3 (four knots) there was no evidence of non-linearity (likelihood-ratio χ² = 0.74, 2 df, p=0.69). All values are produced by the deposited code (03_cox_regression.py, 07_revision_models.py). This table replaces the former Table 2, which duplicated the forest plot.
