# Supplementary material for: Predictors of post-retirement mortality in athletes required to maintain very high body mass: a retrospective cohort study using AI-driven three-dimensional body reconstruction, 1865–1990
Source: PeerJ. 2026 Nov 10;14:e21924. doi: 10.7717/peerj.21924 (PMC13762328; doi:10.7717/peerj.21924)
Supplement: Supplementary file 4 [file peerj-14-21924-s008.docx]

**Supplemental Table S2.** Sensitivity of the reconstructed sagittal diameter, of C3, and of the hazard ratio for C3 to the geometric assumptions of the volume-conserving solver, C3 analysis set (n=587, deaths=450).

| **Assumption (trunk mass fraction; area at maximal girth / mean trunk area)** | **SAD median, cm** | **SAD/TAD** | **C3 median, cm** | **r (C3, BMI)** | **HR for C3 per SD (95% CI), Model A** | **Harrell’s C** |
| --- | --- | --- | --- | --- | --- | --- |
| Primary analysis: mass fraction 0.55, section factor 1.20 | 40.6 | 0.92 | 136 | 0.95 | 1.47 (1.29–1.69) | 0.675 |
| Mass fraction 0.55, section factor 1.30 (circular section) | 43.9 | 1.00 | 141 | 0.96 | 1.46 (1.28–1.67) | 0.675 |
| Mass fraction 0.497, section factor 1.20 | 36.6 | 0.83 | 130 | 0.93 | 1.48 (1.29–1.70) | 0.674 |
| Mass fraction 0.55, uniform trunk (1.00) | 33.8 | 0.77 | 126 | 0.91 | 1.48 (1.28–1.70) | 0.673 |
| General-population values: 0.497, uniform trunk (1.00) | 30.5 | 0.69 | 121 | 0.88 | 1.46 (1.26–1.68) | 0.671 |

The general-population values are a trunk mass fraction of 0.497 and a trunk of uniform cross-section (Dempster, 1955; Drillis & Contini, 1966; Winter, 2009). Absolute values of SAD and C3 depend on the assumption; the hazard ratio per standard deviation of C3 and the discrimination of the model do not. SAD, sagittal abdominal diameter; TAD, transverse abdominal diameter; C3, virtual three-dimensional waist circumference; BMI, body mass index; HR, hazard ratio.
