# Supplementary material for: Predictors of post-retirement mortality in athletes required to maintain very high body mass: a retrospective cohort study using AI-driven three-dimensional body reconstruction, 1865–1990
Source: PeerJ. 2026 Nov 10;14:e21924. doi: 10.7717/peerj.21924 (PMC13762328; doi:10.7717/peerj.21924)
Supplement: Supplementary file 5 [file peerj-14-21924-s009.docx]

**Supplemental Table S3.** Standardised mortality ratios (SMRs), by sub-cohort and reference rate.

| **Sub-cohort** | **n** | **Observed deaths** | **Person-years** | **SMR (JMD)*** | **SMR (single rate)†** |
| --- | --- | --- | --- | --- | --- |
| Primary cohort overall | 604 | 467 | 18,579 | 1.89 (1.72–2.07) | 2.09 (1.91–2.29) |
| Elite-ranked | 259 | 199 | 7,577 | 2.18 (1.89–2.51) | 2.19 (1.90–2.51) |
| Non-elite | 345 | 268 | 11,001 | 1.72 (1.52–1.94) | 2.03 (1.79–2.29) |
| Pre-1950 birth | 465 | 425 | 14,401 | 1.84 (1.67–2.02) | 2.46 (2.23–2.70) |
| Post-1950 (within primary) | 139 | 42 | 4,178 | 2.57 (1.85–3.48) | 0.84 (0.60–1.13) |
| Birth year ≥1970 sensitivity | 163 | 8 | 1,881 | 2.90 (1.25–5.71) | 0.35 (0.15–0.70) |
| Full analytical cohort | 767 | 475 | 20,460 | 1.90 (1.73–2.08) | 1.93 (1.76–2.12) |

* Primary SMR analysis: expected deaths computed by per-wrestler integration of age × calendar-year-stratified Japanese male reference mortality rates from the IPSS Japan Mortality Database (JMD, 1947–2024), with Gompertz extrapolation for pre-1947 periods. † Sensitivity analysis using a single reference rate of 0.012 per person-year, presented for transparency; the inversion of the post-1950 and birth year ≥1970 estimates illustrates how a single-rate reference fails to account for the secular decline in Japanese male mortality. 95% confidence intervals by the Byar approximation. This table contains the numerical results formerly presented as Table 3, which duplicated the forest plot (Figure 5).
