# Supplementary material for: Predictors of post-retirement mortality in athletes required to maintain very high body mass: a retrospective cohort study using AI-driven three-dimensional body reconstruction, 1865–1990
Source: PeerJ. 2026 Nov 10;14:e21924. doi: 10.7717/peerj.21924 (PMC13762328; doi:10.7717/peerj.21924)
Supplement: Supplementary file 6 [file peerj-14-21924-s010.docx]

**Supplemental Table S4.** Documentation of cause of death by period of death and elite rank, primary cohort (467 deaths).

| **Period of death** | **Non-elite: documented / deaths** | **Elite: documented / deaths** |
| --- | --- | --- |
| to 1926 (Meiji, Taishō) | 0/7 (0%) | 2/4 (50%) |
| 1927–1945 (pre-war Shōwa) | 0/36 (0%) | 11/29 (38%) |
| 1946–1969 | 2/59 (3%) | 16/47 (34%) |
| 1970–1989 | 9/85 (11%) | 36/44 (82%) |
| 1990 onward | 46/81 (57%) | 69/75 (92%) |
| All periods | 57/268 (21%) | 134/199 (67%) |

Values are the number of deaths with a documented cause over the number of deaths (percentage). Elite rank: highest rank attained was yokozuna, ōzeki, sekiwake, or komusubi. No cause was documented for any non-elite death before 1946, and a cause was documented for more than half of deaths in both rank groups only from 1990 onward; cause-specific analysis was therefore restricted to follow-up from 1990.
